# Bilingualism modulates functional connectivity induced by a domain-general artificial grammar learning task

**DOI:** 10.1038/s41598-026-42094-x

**Published:** 2026-03-09

**Authors:** Alex Sheehan, Doug Saddy, Diego Krivochen, Shruti Gupta, Mickey Sibsey, Christos Pliatsikas

**Affiliations:** 1 School of Psychology and Clinical Language Sciences, University of Reading, Reading, UK; 2 School of Languages, Linguistics, Literatures and, Cultures, Faculty of Arts, University of Calgary, Calgary, AB Canada; 3 King’s College Hospital NHS Foundation Trust, London, UK; 4 Facultad de Lenguas y Educación, Centro de Ciencia Cognitiva, Universidad Antonio de Nebrija, Madrid, Spain

**Keywords:** Neuroscience, Psychology, Psychology

## Abstract

Bilingualism is associated with distinct patterns of resting-state functional brain connectivity – a consequence of ongoing language control demands that do not apply to monolinguals. However, it is not well understood how these patterns affect, and are affected by, brain activation for domain-general cognitively demanding tasks. Here, we employ a novel task-driven resting-state electroencephalography design including an implicit Lindenmayer grammar learning task, which tracks aperiodic and hierarchical dependencies, to determine task-related functional connectivity changes in bilinguals. Quantified bilingual experience was used as a predictor of directional effects, using Generalised Additive Models to account for non-linear patterns. Our results revealed post-task alterations to connectivity involving increased involvement of occipital regions, reduced involvement of frontal and central regions, and faster reaction times to stimuli at higher levels of bilingual experience. Crucially, the regions implicated post-task appear to reflect task-relevant regions which are involved in the language and executive control networks, reflecting greater short-term task-driven flexibility. These findings have important implications for our understanding of how domain-general processing and connectivity are shaped by linguistic experience.

**Supplementary Information:**

The online version contains supplementary material available at 10.1038/s41598-026-42094-x.

## Introduction

Artificial grammar learning (AGL) paradigms have long been used to assess implicit statistical learning in psychological research^1^, providing insights into how the brain detects patterns within the mass of information input from our senses. This includes evidence suggesting that performance in AGL tasks is correlated with performance on word predictability tasks^2^ and language comprehension^3^. More recent explorations have used AGL paradigms to determine whether language utilises domain-specific or domain-general cognitive functions to process sequential inputs. In a study comparing individuals with developmental language disorder (DLD) to a neurotypical control group, the DLD group displayed significantly lower learning rates in verbal AGL and non-verbal motor sequence tasks, with no differences between DLD and controls in a non-sequential probabilistic categorisation task^4^. Similar results have been found in populations with agrammatic aphasia^5^ and dyslexia^6^, indicating a deficit of sequential learning in language disorders generally, but no deficit in non-sequential learning. This suggests that the functions supporting language processing as sequentially presented information are domain-general rather than language-specific.

The above findings raise an important question: if sequential learning strongly correlates with language ability, and if language impairments result in implicit statistical learning deficits, how is sequential learning affected by language expertise and experience? Bilinguals and multilinguals (henceforth “bilinguals”) constitute a group with unique and multifaceted language experiences, having acquired and gained experience in managing multiple language representations^7^. In language processing tasks, bilinguals have shown worse performance than monolinguals: smaller receptive vocabularies^8^, lower scores on picture naming tasks^9^, and lower comprehension accuracy in ambiguous sentence processing^10^. Conversely, some studies have shown bilinguals to have greater processing abilities than monolinguals for non-linguistic stimuli, including greater accuracy and shorter reaction times than monolinguals on inhibition tasks, such as Flanker and Simon tasks^11–13^, reduced incongruency effects associated with earlier second language (L2) ages of acquisition (AoA)^14^, greater task-switching performance^15^, and greater memory abilities^16^. However, these effects have not always been replicable^17,18^. These domain-specific studies indicate that bilingualism can have significant effects on cognition, affecting both the speed and efficiency of processing, modulated by aspects of language experience.

Bilinguals are well-practiced at extracting information from strings corresponding to structures with differing rules and constraints, as they must understand multiple languages with different patterns and regularities^19,20^. This observation aligns with the Structural Sensitivity Theory^21^, which states that exposure to diverse structures makes deviations and similarities in structures more salient to bilinguals, and hence better able to identify and respond to them. This experience in processing linguistic structures represents an opportunity to observe how increased exposure to strings generated by different rule systems (‘grammars’) affects the processing of other non-linguistic strings generated by different grammars. Previous AGL studies have found bilinguals to exhibit reduced reaction times^22^ and greater sensitivity to grammatical errors compared to monolinguals, even at just 24-months of age^23^.

However, these studies used the outputs of grammars which follow consistent periodic patterns, whereas bilinguals’ grammatical inputs stem from multiple language sources with different structures and levels of aperiodicity. Such canonical grammars and the periodic strings they generate are less complex than the natural languages typically experienced in everyday life^24,25^. Typical AGL designs use strings generated from a starting symbol by ‘rewriting rules’, which determine how the symbol can be rewritten to generate a string (e.g., X -> Y, ‘rewrite X as Y’). These grammars contain rewritable and non-rewritable symbols^26^: when the rules deliver a string of non-rewritable symbols, the rewriting process terminates. Rules in these grammars must apply in a certain order (a ‘traffic convention’). Participants are typically exposed to strings generated by a grammar, and then required to determine whether new strings were also generated by the same grammar or respond to the same regularities^27^. An issue with this method is its reliance on implicit knowledge gained from exposure to the outputs of the grammar becoming explicit when judging the strings, meaning that these tasks may lose sensitivity to the implicit learning processes taking place.

To overcome this problem, we utilised a different class of artificial grammars known as Lindenmayer grammars (L-grammars)^28^. These types of grammars are unique in their properties: firstly, they make no distinction between rewritable and non-rewritable symbols (i.e. a symbol which terminates the generation of a sequence). Secondly, there is no sequential ‘traffic convention’ (specific order of application) for the rewrite rules: all symbols are rewritten simultaneously in a top-down, fractal-like fashion. Thirdly, the structures generated by these grammars are self-similar, meaning that the superficial properties of each generation of the grammar will map hierarchically onto a previous generation. This allows for non-adjacent generations of the grammar to be reconstructed based on a limited sample of the sequence. Crucially, strings generated by L-grammars contain statistical regularities which the experimenter can control. This paper will use an L-grammar known as the Fibonacci grammar (henceforth “Fib”) – so called because the number of symbols in each generation of the grammar, as well as the number of occurrences of each symbol, will always be a Fibonacci number. Fib has only two rules:

$${\text{Rule A}}:{\text{ }}0{\text{ }} \to {\text{ 1}}$$

$${\text{Rule B}}:{\text{ 1 }} \to {\text{ }}0{\text{ 1}}$$

As a consequence of the application of these two rules, two deterministic regularities and one probabilistic regularity follow in any generated string: a 0 will always be followed by a 1 and two 1s will always be followed by a 0 – these are referred to as ‘unambiguous’ points. The probabilistic regularity is that a 1 can be followed by either a 0 or a 1 – referred to as ‘ambiguous’ points. These points can be disambiguated by considering the hierarchical structure derived by the grammar. Due to the self-similarity of the grammar, transitional properties of the individual items at each hierarchical level remain identical, allowing symbols in the output string to be grouped into larger constituents (e.g., [01]), whilst the probabilistic transitions of constituents at one hierarchical level are recursively embedded into deterministic transitions at the next. This allows individuals to bypass the typical limits of human working memory by chunking the string into constituents, constructing the hierarchy of the sequence without direct access to the rules. The aperiodicity of the string also aids this approach by reducing habituation effects, which traditional AGL paradigms rely on for error detection during grammaticality judgements due to increased salience^29^. The aperiodicity of the Fib strings means that this approach would lead to incorrect predictions of the next item, and so an alternative approach must be used. The preservation and recursive embedding of transitional properties in constituent chunking delivers a hierarchical model which embeds self-similar constituents, such that future items can be predicted based on transitional properties of previous generations without necessitating habituation. We provide a more thorough description of L-grammars, their properties, and utility compared to the grammars used in typical AGL designs in the Supplementary Materials. For a comprehensive account of the Fibonacci grammar’s properties, including its self-similarity, aperiodicity, and hierarchical recursive embedding, see Krivochen^30^, and for reviews and experimental evidence showing how L-grammars’ unique properties can be used in cognitive tasks to differentiate and measure sequential and hierarchical learning, see Schmid et al. and Vender et al.^31–33^.

This grammar has been used successfully in behavioural studies to assess implicit statistical learning, similarly to previous AGL designs, but its hierarchical mapping of surface-level transitional properties additionally necessitates hierarchical structure processing^31,34,35^, as natural language does^36^. Given the impact of bilingualism on cognition, with faster reaction times and greater accuracy observed in executive function tasks^11,13,15,37^ and faster reaction times on AGL tasks with higher sensitivity to grammatical errors and inconsistencies^22,23^, it is expected that language experience would affect performance on AGL tasks involving L-grammars, as it does for traditional canonical grammars. Neuroimaging studies conducted using AGL tasks have noted increased activation of central and temporal brain regions^38–40^– also involved in the language and frontoparietal executive control networks^41–44^, further suggesting that bilingual experience may impact the neural response to AGL tasks and affect behavioural performance. To test the impact of bilingualism on processing the Fib grammar, Vender and colleagues^33^ adapted a Simon task, which utilises congruency and incongruency between stimulus and response locations to assess inhibitory control and executive function^45^. The task used red and blue squares as stimuli, representing the 0s and 1s of the Fib grammar respectively, to form a serial reaction time task^33^. Upon administering this task to bilingual and monolingual dyslexic and non-dyslexic children, they found that the bilingual non-dyslexic group exhibited faster reaction times (RTs) than monolingual non-dyslexics, bilingual dyslexics outperformed monolingual dyslexics, and in some cases, bilingual dyslexics performed at the same level as monolingual non-dyslexics. A similar study presented all stimuli centrally, removing the incongruency of the Simon task^32^. This version of the task dispensed with the demand of additional executive control resources and allowed for a closer analysis of the learning that takes place. Results showed that participants’ RTs decreased as they progressed through the hierarchical levels of the output string for both ambiguous and unambiguous trials. Critically, this decrease was greater for unambiguous points at higher hierarchical levels, indicating that constituent chunking and hierarchical structure processing were employed to predict subsequent stimuli. This version of the task is particularly suitable as a domain-general cognitive task – with the potential executive function confound removed, the task demands constitute only implicit statistical learning and hierarchical structure processing. These abilities are utilised in many cognitive processes across domains and modalities, representing generalised cognitive abilities^46–48^.

From the above evidence, it is clear that behaviourally, bilingualism has a significant effect on hierarchical structure processing and implicit statistical learning, consistent with evidence of bilingual adaptations to cognition, particularly in the language and executive function domains^49^. Notably, these behaviourally observed cognitive effects have also been associated with adaptations to brain function in bilinguals. For example, during inhibition tasks, bilinguals rely more heavily on bilateral subcortical, frontal, and temporal brain regions compared with greater left hemisphere temporal and parietal activation in monolingual participants as measured using functional magnetic resonance imaging (fMRI)^50^. Moreover, using magnetoencephalography to record brain activity during an inhibition task, Bialystok and colleagues^11^ found that faster reaction times on the task were associated with greater activation in left hemisphere frontal, temporal, and cingulate regions – but only for the bilingual participants, with no such relationship for the monolinguals. These studies show differing brain responses between bi- and monolingual individuals for the same tasks, suggesting that previously reported behavioural differences may be driven by differences in brain function and neural recruitment strategies. Despite these findings, the neural mechanisms which support increased bilingual performance on implicit statistical learning tasks remain unexplored.

As previously stated, bilingualism has been found to not only affect how stimuli are processed, but the way the brain communicates internally. The Bilingual Anterior to Posterior and Subcortical Shift (BAPSS) model^51^ postulates that as bilinguals gain language experience, they exhibit an enhancement in the efficiency of brain region recruitment and connectivity. This is associated with a shift from relying primarily on frontal and prefrontal structures to relying more on posterior and subcortical regions – consistent with results showing greater subcortical recruitment for bilinguals compared to monolinguals during tasks requiring inhibition^50^. Resting-state electroencephalography (rs-EEG) studies have provided additional support for this model, finding greater signal complexity (as measured using multiscale entropy, a measure of uncertainty) – typically associated with better processing abilities^52,53 ^– in posterior electrodes for bilingual participants compared to monolinguals^54^. Other studies have utilised functional connectivity analyses to assess the impact of bilingualism on brain function. For example, Bice and colleagues^55^ compared bi- and monolinguals in an rs-EEG study using the connectivity metric of coherence, which determines correlations between activity in different regions. This type of analysis splits the signal into distinct frequency bands, each of which is associated with different cognitive functions. Bice and colleagues reported greater connectivity for bilinguals versus monolinguals between posterior regions and frontal, fronto-temporal, and parietal regions. Notably, the majority of the significant bilingualism-modulated connections were found in the frequency bands associated with concentration and anxiety (beta), and attentional and inhibitory processes (alpha)^56^. These results support the conclusions of the BAPSS model and demonstrates that bilingualism significantly impacts signal complexity and connectivity in posterior regions in multiple frequency bands of activity.

A similar rs-EEG study was conducted by Pereira Soares and colleagues^57^, who utilised an all-bilingual sample and measured aspects of their linguistic experience continuously using a language history questionnaire. This approach allowed for a more nuanced interpretation of the impact of linguistic experience, instead of compounding individuals with vastly different levels of language experience into a single ‘bilingual’ group^58^, directly addressing the predictions of the BAPSS model^51^, which notes that the cumulative increase in language *experience* drives functional adaptations. Pereira Soares and colleagues found that the strength of interhemispheric posterior connections was modulated by L2 AoA and non-societal language exposure at home in the alpha and gamma frequency bands. The results also showed that bilingual experience affects frontal connectivity, with proficiency in the societal language and exposure to a non-societal language in the community significantly impacting connections in the theta band of activity, typically associated with long-distance neural communication and working memory^59^, as well as the alpha band. Following up, Voits et al.^60^also used an all-bilingual sample but with a large age range, in order to investigate potential interactions between bilingual experience and age on functional connectivity patterns. Voits and colleagues used a measure of bilingual experience defined as Multilingual Language Diversity (MLD); a ratio of language usage calculated using Shannon entropy, a measure of uncertainty. The higher the MLD score, the more diverse the individual’s usage of languages. Their results showed significant non-linear interactions between the two variables of interest (MLD, age) and frontal, fronto-temporal, and occipital region connectivity, including both inter- and intra-hemispheric effects in the alpha, beta, and theta bands. The results of the experiment determined that a higher MLD score compensates for some age-related decline in connectivity and signal power, reflecting preserved brain function in the face of cognitive ageing. Whilst previous rs-EEG studies had relied solely on linear statistics, this study importantly revealed that some of the relationships between bilingualism and functional connectivity are non-linear in nature.

These studies strongly support the BAPSS model, showing greater connectivity between occipital regions and the rest of the brain with increasing bilingual experience, including long-distance and inter-hemispheric connections. However, these studies utilise only *resting*-state functional connectivity – the brain’s intrinsic baseline state when not engaged in active cognition, meaning that this activity cannot be linked to specific cognitive functions^61^. What remains unknown is how the bilingual brain shifts from this baseline when encountering specific cognitive demands. To overcome this limitation, we employed a technique known as *task-driven resting-state*. This method records participants’ resting-state activity both pre- and post-task, allowing comparisons between the baseline and the brain state induced by the task. Although this technique has been used in fMRI and EEG research^62–64^, to our knowledge, it has never been utilised in bilingualism research. Whilst the structure of resting-state functional networks has been found to be stable over the course of years^65^, these networks can flexibly adapt to exogenous and endogenous influences and reorganise based on cognitive demand, showing short-term variability over the course of minutes^66^. An advantage of task-driven resting-state designs over using on-task recordings for functional connectivity analysis is that they avoid the confounding impact of overlapping, simultaneous task-evoked activity^67^, and instead provide an offline view of the global short-term reorganisation of connectivity induced by the task’s cognitive demands – a lasting ‘after-effect’ of the task^68^. This technique represents an ideal method of investigating the interaction between language experience and task demands, and its impact on short-term functional reorganisation. This allows one to probe both the long-term adaptations to connectivity associated with bilingual experience using the pre-task data, and the bilingualism-modulated short-term adaptations to connectivity in response to an exogenous influence using the post-task data. Additionally, the task-driven technique provides a cognitive anchor to link any changes in connectivity – by administering tasks with specific experimenter-controlled cognitive demands, one can determine exactly how the brain responds. Post-task, previous studies have found increased brain signal complexity^69^, reflecting a change in brain state. Typically, pre-task connectivity reflects more widespread intrinsic activity, followed by a shift to more localised task-specific activity post-task^63,70,71^. By incorporating aspects of both task-based and task-free designs, one can construct more detailed interpretations of how task demands impact the brain’s functional architecture, as well as the role which neuroplastic experiences play in adapting this architecture.

The present study utilised a novel task-driven resting-state EEG design to assess the impact of level of bilingualism on modulations to the network dynamics of the brain related to domain-general cognitive demands. To obtain a continuous measure of bilingual experience, we used the composite score provided by the Language and Social Background Questionnaire (LSBQ)^72^. We used a whole-brain EEG approach with Granger Causality as the connectivity metric – which is directional and therefore shows the flow of information in the brain, allowing for a comprehensive overview of regional connectivity, and a close examination of the directional predictions of the BAPSS model^51^. Due to the non-linear nature of structural^73^ and functional^60^ adaptations conferred by bilingualism, we used Generalised Additive Models^74^ for our statistical analysis, allowing for linear and non-linear relationships to be captured.

Whilst this analysis was largely exploratory, some rough predictions as the nature of the connectivity patterns that may emerge were formed. In the pre-task condition, we expected to observe long-distance connections modulated by level of bilingualism between frontal and occipital regions, possibly also with connections between the above regions and temporal regions^55,57^. We expected the pre-task connectivity pattern to include connections in both hemispheres, and that the flow of information will be directed posteriorly, as predicted by the BAPSS model^51^. For the post-domain-general-task condition, we predicted general bilingualism-modulated connectivity between occipital and more frontal regions, again with the flow of information directed towards occipital regions due to the more distributed processing predicted in the BAPSS model. Based on previous neuroimaging of statistical learning tasks, we also expected to observe increases in connectivity strength in central and temporal regions^38–40^ as overlapping regions involved in both implicit statistical learning and the language network^41,42^.

## Results

### Behavioural

During the task, accuracy was consistently high (mean = 0.97, SD = 0.002), and consistent reaction time decreases were found with increased exposure to the sequence (mean change = -83.888 ms), suggesting that learning and prediction of the sequence took place. The behavioural models are split by ‘level of ambiguity’ – this term refers to the hierarchical level at which participants processed and extracted transitional probabilities from the sequence. For example, ambiguity level 0 represents surface level processing of transitional probabilities between the individual 0s and 1s of the sequence. These individual items are then chunked into larger groups, allowing for ambiguous points at the surface level to become disambiguated due to the grammar’s aperiodicity and self-similarity. Ambiguity level 1 is the first of these ‘chunked’ levels. The initial constituent chunks are then grouped even further based on the transitional probabilities between them, allowing for further ambiguous points to be disambiguated and subsequently predicted, with the level of ambiguity increasing each time, representing the number of levels of hierarchy in the sequence’s structure that the individual was able to process. Below, we present a selection of the behavioural results to illustrate the learning effects which took place. The full results of the behavioural analysis can be found in the Supplementary Materials.

**Fig. 1:**
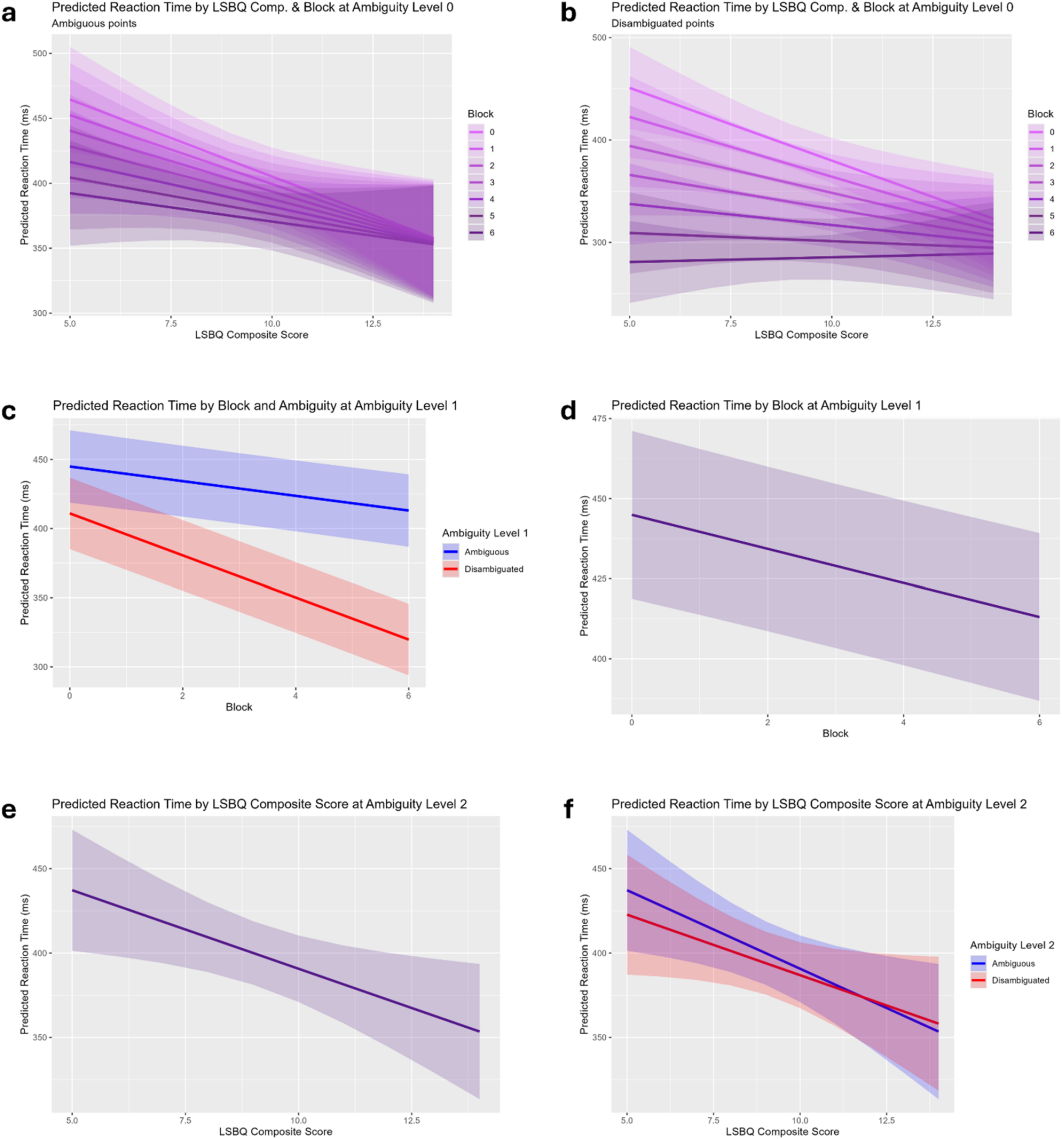
Example plots from the behavioural data analysis. (**a**) Mean reaction time in each block by LSBQ composite score for ambiguous points at ambiguity level 0. (**b**) Mean reaction time in each block by LSBQ composite score for disambiguated points at ambiguity level 0. (**c**) Reaction time by block for ambiguous and disambiguated points at ambiguity level 1. (**d**) Overall reaction time by block at ambiguity level (1) (**e**) Overall reaction time by LSBQ composite score at ambiguity level (2) (**f**) Reaction time by LSBQ composite score for ambiguous and disambiguated points at ambiguity level 2.

At ambiguity level 0 (processing surface-level statistical regularities), there is evidence of faster reaction times by those with greater bilingual experience (*β* = -11.861, *p* < 0.01), among other significant effects. The results from ambiguity levels above zero (processing hierarchical regularities) show that with increasing bilingual experience, participants exhibited flatter learning slopes across blocks at all levels of ambiguity (e.g. Level 1: *β* = 1.041, *p* < 0.01; Level 4: *β* = 2.746, *p* < 0.001). Individuals with higher levels of bilingual experience also showed faster reaction times generally (e.g. Level 2: *β* = -14.616, *p* < 0.001). These findings are illustrated by the example plots of significant terms in the models from ambiguity levels 0, 1, and 2, shown in Fig. 1.

**Table 1:** Models in which granger causality is significantly modulated by level of bilingualism in the pre- and post-task conditions after FDR correction.

		Region to:			
Pre-task:		Medial frontal	Medial occipital	Right central	Right parietal
Region from:	Left central			*	**
	Medial occipital	**			
	Right central		***		
	Right temporal				**

		Region to:				
Post-task:		Left central	Left parietal	Left temporal	Medial occipital	Right parietal
Region from:	Left central			*	*	***
	Left temporal		*			
	Medial frontal	***		*	***	

Asterisks indicate *p*-value: * = <0.05, ** = <0.01, *** = <0.001.

### Pre-task

**Fig. 2:**
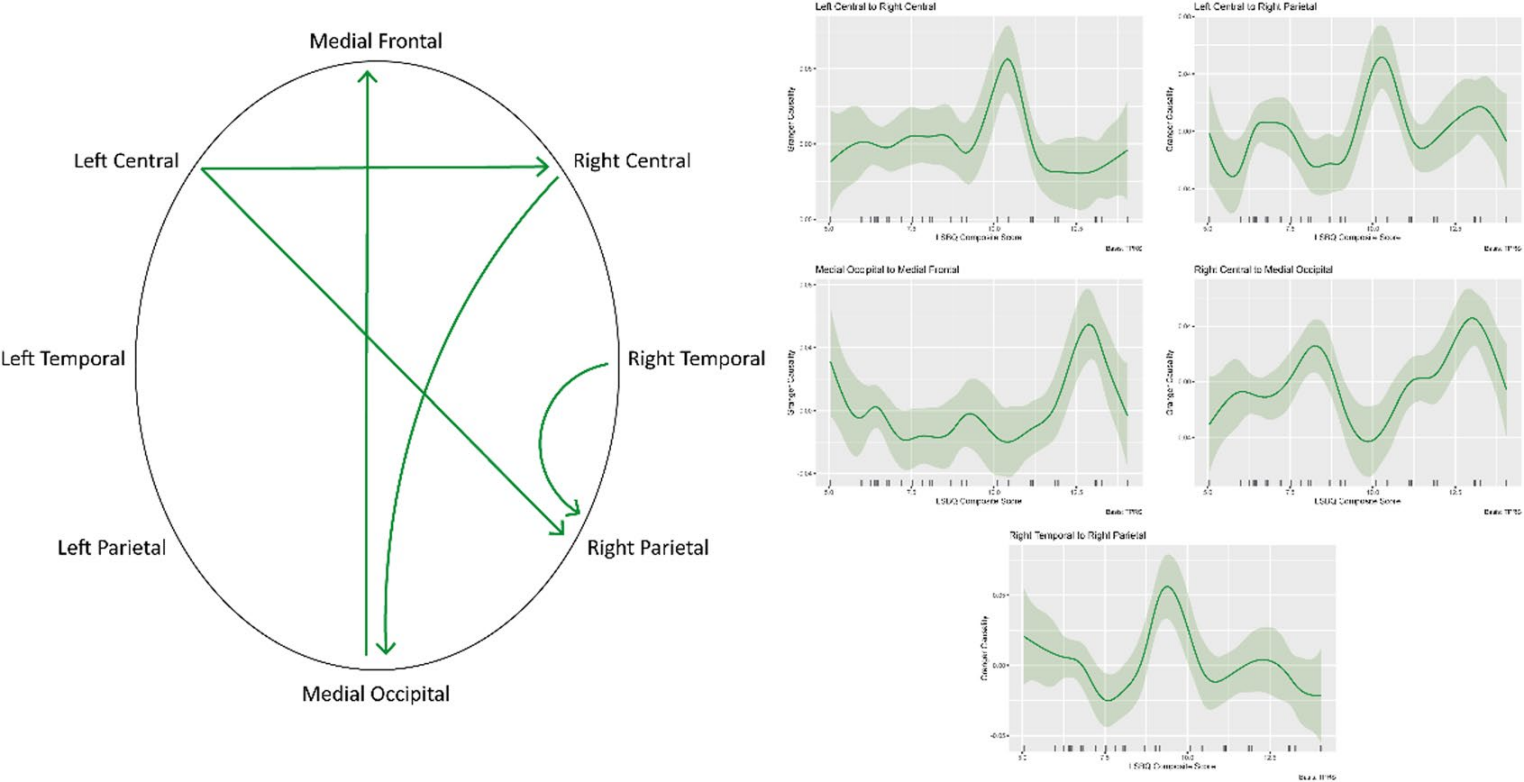
The results of the pre-task functional connectivity analysis. The left side displays the connections significantly modulated by level of bilingualism on a head diagram. Arrows indicate the direction of the flow of information. The right side shows the partial effects plots of the model smooths, showing LSBQ composite score on the X axis and Granger Causality value on the Y axis. Please note that the Y axes of each plot vary as they are scaled to best visualise the relationship. The shaded areas above and below the smooths show credible intervals.

The pre-task condition revealed five connections significantly modulated by level of bilingualism, shown in Table 1. All show variable non-linear relationships between bilingual experience and functional connectivity, with three of the significant connections (left central to right central, left central to right parietal, and right temporal to right parietal) showing similar spikes in connectivity strength at mid-levels of bilingual experience (an LSBQ composite score of ~10). The connections are visualised on an electrode montage diagram with plots of the smooths in Fig. 2.

### Post-task

**Fig. 3:**
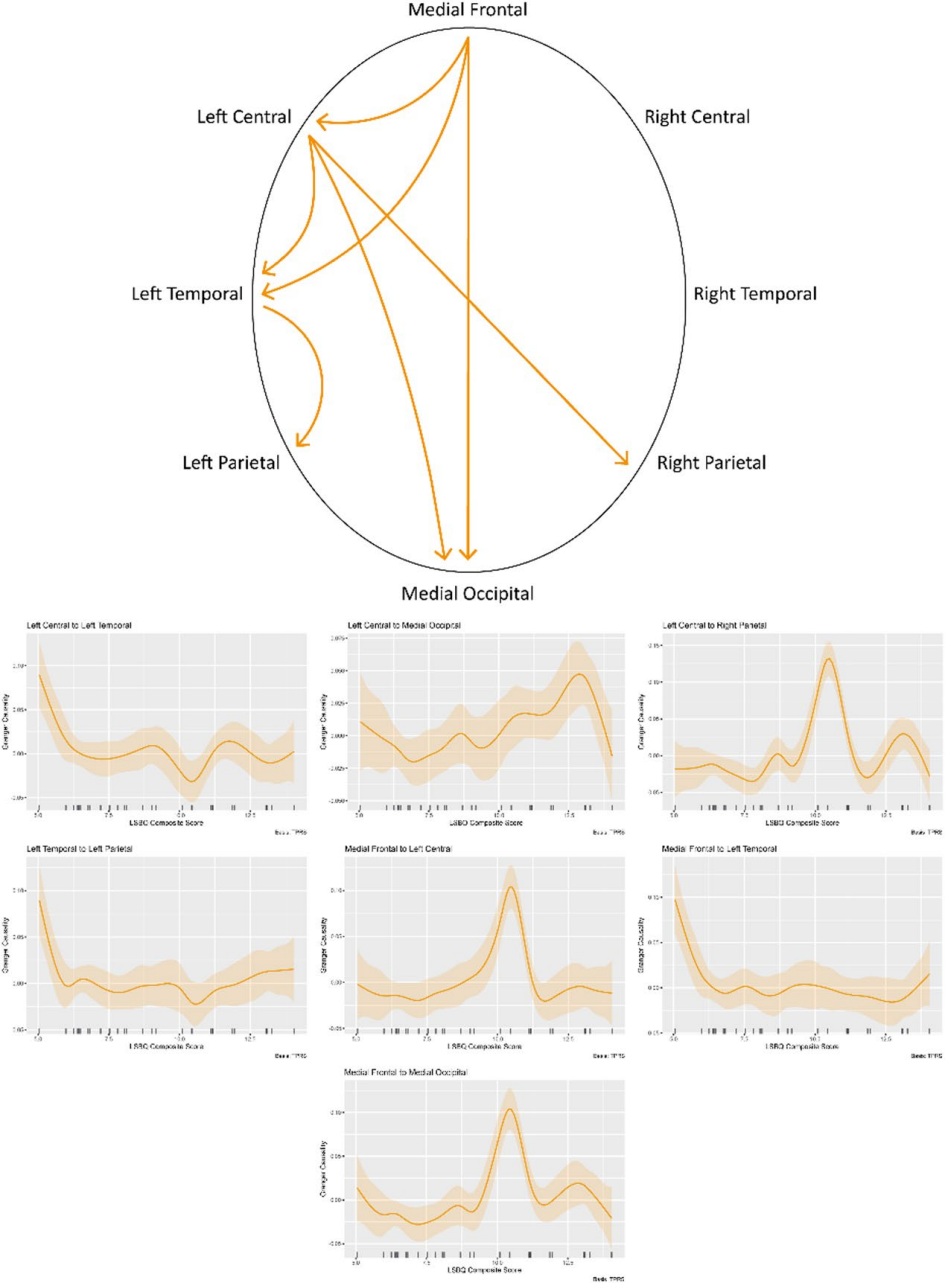
The results of the post-task functional connectivity analysis. The upper side displays the connections significantly modulated by level of bilingualism on a head diagram. Arrows indicate the direction of the flow of information. The lower side shows the partial effects plots of the model smooths, showing LSBQ composite score on the X axis and Granger Causality value on the Y axis. Please note that the Y axes of each plot vary as they are scaled to best visualise the relationship. The shaded areas above and below the smooths show credible intervals.

In the post-task condition, seven connections were found which showed significant effects of level of bilingualism, detailed in Table 1. Similarly to the pre-task condition, all showed variable non-linear relationships, but of differing morphology compared to that of the pre-task connections. Specifically, three of the significant connections also exhibited spikes in connectivity strength at LSBQ scores of ~10 (left central to right parietal, medial frontal to left central, medial frontal to medial occipital). The medial frontal region here shows three outbound significant connections – a shift from the solely inbound medial frontal connection found pre-task. These connections can be seen on an electrode montage diagram above plots of the smooths in Fig. 3.

**Fig. 4:**
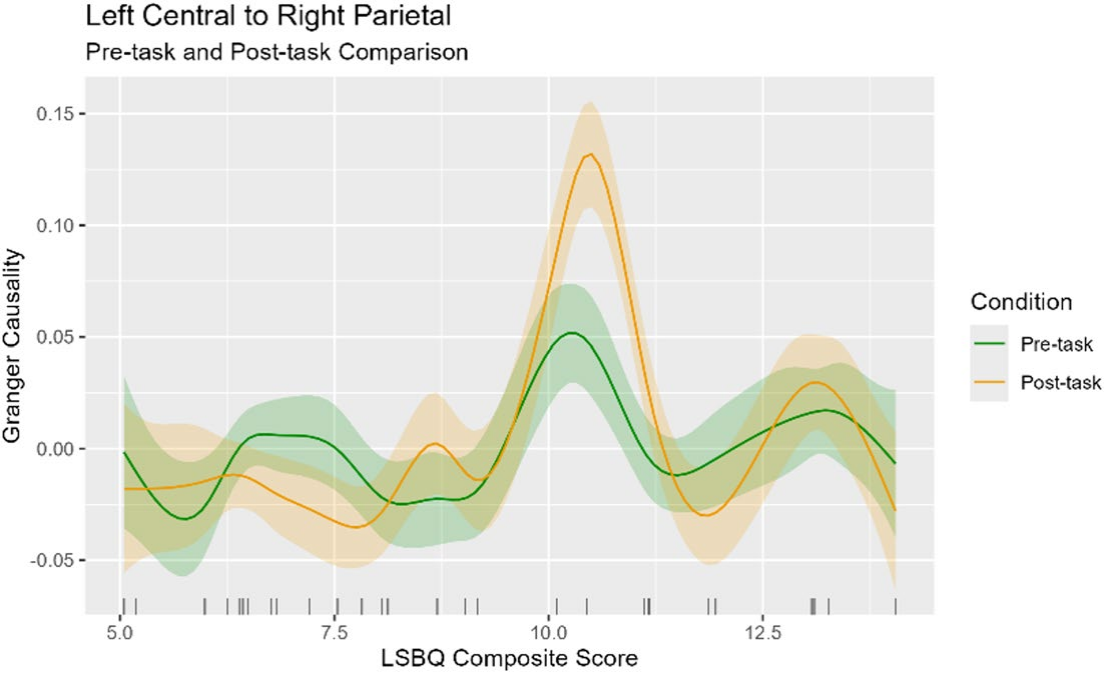
A comparison of the left central to right parietal connection between the pre- and post-task conditions. The partial effects plot shows LSBQ composite score on the X axis, and Granger Causality value on the Y axis. The pre-task smooth is shown in green, and the post-task smooth is shown in orange. The shaded areas above and below the smooths show credible intervals.

In both the pre- and post-task conditions, the left central to right parietal connection emerged as being significantly modulated by LSBQ composite score. In both cases, it shows an increase in connection strength at an LSBQ score of around 10. However, in the post-task condition, this increase is roughly double the size of the one observed in the pre-task condition. For comparison, this is shown on a dual-smooth partial effects plot in Fig. 4.

## Discussion

This study revealed which functional connections were modulated by degree of bilingual experience following a domain-general cognitive task involving implicit statistical learning and hierarchical structure processing when compared to a baseline resting-state recording. This was the first study to use a task-driven resting-state design on a bilingual sample, and the first to examine the neurological underpinnings of the Fib grammar task. Critically, we used a gradient approach to bilingualism – measuring experience continuously without the need for separate language groups, paired with non-linear statistical analysis and a directional connectivity measure, allowing for a closer examination of the effects of bilingualism on brain function.

The results of the pre-task condition showed bilingualism-modulated long-distance and interhemispheric connections across the brain – specifically, involving frontal, occipital, central, temporal, and parietal regions, primarily in the right hemisphere. The significant connections which involved the medial occipital region both exhibit a peak in connection strength at higher levels of bilingual experience, whereas the remaining connections show peaks at mid levels of experience. This observation is compatible with the BAPSS model’s prediction of stronger connectivity in posterior regions with increasing bilingual experience^51^, particularly as the connections involving the left and right central regions displayed stronger connectivity at mid levels of experience, showing the previously-observed pattern of highlighting a possible shift from anterior to posterior reliance as language experience increases. Whilst the information flow in the pre-task condition was mainly from anterior to more posterior regions, the single outbound connection from the medial occipital region was directed towards the medial frontal region – contrary to our prediction. However, some previous evidence does exist of stronger fronto-occipital connectivity in bilinguals compared to monolinguals^75^, of which this connection could be a reflection due to the peak connectivity strength occurring at higher levels of experience. Generally, the regions implicated in our analysis are consistent with previous rs-EEG connectivity studies conducted on bilinguals, which found connections significantly associated with bilingual experience in frontal, posterior, and fronto-temporal regions^55,57,60^. Our electrode montage further separated the brain regions analysed to distinguish between temporal, parietal, and medial occipital regions, as opposed to the medial frontal, left and right fronto-temporal, and left and right posterior regions used in the previous investigations. Consequently, the connectivity patterns observed in this study exhibit some differences whilst still showing similar overall effects in frontal and posterior regions, including a bilingualism-modulated connection between (in our montage) left and right central regions, and (in previous studies’ montages) left and right fronto-temporal regions.

In the post-task condition, a shift in connectivity pattern can be observed – contrary to the pre-task results, this condition showed primarily left hemisphere connections modulated by bilingual experience level. Though, similarly to the pre-task condition, the connections which emerged as significant are long-distance, and involve frontal, occipital, central, temporal, and parietal regions. The medial frontal and left central regions each show three outbound connections, which could be interpreted as task-relevant ‘hubs’ for stimuli processing. This is consistent with previous neuroimaging studies, which have found that the prefrontal cortex is strongly associated with processing hierarchical structure, particularly when working memory demands are high^38–40^. The behavioural results of the task show faster reaction times at higher levels of bilingual experience across all levels of ambiguity, suggesting that more experienced bilinguals were able to extract the transitional probabilities and process the hierarchical structure of the sequence earlier and more efficiently than those with lower experience. This may then suggest that more highly experienced bilinguals exhibited less reliance on connections between frontal and central regions for higher levels of ambiguity, and instead displayed greater connectivity with the medial occipital region. This view is consistent with the anterior-to-posterior shift described in the BAPSS model, but to more concretely provide support for this claim, a study using a similar task conducted under fMRI, for higher spatial resolution, would be necessary.

Crucially, the post-task results show only posteriorly-directed connections as significantly modulated by bilingual experience – consistent with the BAPSS model’s predictions. As the left central region of our montage was located over the prefrontal cortex, which is an essential region for both hierarchical structure processing and domain-general cognitive control^39,43^, the connection between the left central and medial occipital regions of our montage is of particular interest. This connection exhibits its strongest connectivity at high levels of bilingual experience, which could be indicative of the shift from anterior to posterior reliance described by the BAPSS model, particularly due to the faster reaction times shown by participants with higher levels of bilingual experience. This pattern suggests more efficient task performance with lower frontal and central connectivity with other non-occipital regions relative to those with lower experience. Whilst we do not consider this finding confirmatory, the BAPSS model notes that in cases where bilinguals recruit frontal regions more heavily, they often show reduced task performance, whereas here, we observed the opposite pattern. Future studies should pay particular attention to directed prefrontal to occipital functional connections in different cognitive contexts, as it may be particularly useful for confirming under which conditions the BAPSS model’s predictions apply.

There emerged only one connection which was significant in both the pre- and post-task conditions; the left central to right parietal connection. The relationship between LSBQ composite score and connection strength exhibits strong similarities between the two conditions, but with the post-task condition possessing a much larger peak in strength at mid levels of experience. The right hemisphere parietal region has previously been implicated in mathematical processing^76^, specifically, numerical ordering and symbolic arithmetic^77^. The right parietal region’s activation has also been found to reflect learning in a probabilistic task^78^, and is essential for temporal attention^79^. Moreover, the region is a part of the frontoparietal executive control network, which previous studies have revealed to show greater efficiency in bilinguals compared to monolinguals^80^, and exhibits spatial overlaps with regions of the language network^81^. Based on previous evidence, it seems likely that in our sample, the post-task increase in connection strength reflects the cognitive and energetic cost of developing predictions as to how the sequence will proceed through hierarchical analysis of the observed sequence’s structure. As the peak connection strength occurs at mid levels of bilingual experience, this may also indicate that this region is required more heavily by those with less efficient rule extraction during the task. Those with higher bilingual experience may utilise this region to a lesser extent throughout the task due to greater efficiency in learning transitional probabilities, illustrated by lower initial reaction times. Further studies using fMRI or EEG source localisation with an L-system task would provide a more thorough investigation into this interpretation, particularly with the addition of a non-hierarchical statistical learning task as a control, allowing for differentiation between regions utilised for learning and processing transitional probabilities, and those utilised for hierarchical structure processing.

In summary, the present study was the first to reveal short-term bilingualism-modulated alterations to functionalconnectivity driven by task demands. The pre-task condition exhibited a larger number of right hemisphere connections as being modulated by bilingual experience, whereas post-task, this pattern flipped, revealing a greater proportion of left hemisphere bilingualism-modulated connections. The pre-task results follow the general trends observed in previous rs-EEG studies with bilingual samples, and show the more distributed connectivity profile predicted by previous task-driven resting-state studies. The shift from modulations of right hemisphere connections pre-task to left hemisphere connections post-task may be the result of bilingual adaptation to language- and control-related regions. Bilingualism necessitates higher levels of language control than for monolinguals, owing to the fact that in order to use the appropriate language for a given context, the context-inappropriate languages must be suppressed^82^. These additional cognitive demands require greater recruitment of the language and frontoparietal executive control networks^83^, and thus the left hemisphere dominant post-task connections may reflect greater modulation of language- and control-related regional connections amongst bilinguals due to increased efficiency and flexibility.

The present study did, however, face some limitations. The use of EEG as a neuroimaging technique has poor spatial resolution, meaning that interpretations as to which specific areas of the brain were active are limited. Future investigations should consider utilising more spatially accurate techniques such as fMRI, fNIRS, MEG, or EEG with source localisation (which would require a larger electrode array than used in this study). It is also important to note that Granger Causality as a connectivity metric is particularly susceptible to volume conduction, reducing spatial specificity, which should be considered when designing future rs-EEG studies. By using the techniques listed above, one could further localise the neural correlates of the Fib grammar task, which appears to be linked to language experience both in behavioural performance and neurophysiological responses. To further improve analyses investigating the neural underpinnings of the task, future studies should include a non-visual control task and non-grammar-based serial reaction-time task as a method of ensuring that any effects observed are truly the result of the properties of the grammar itself. It is also possible that the location of the study affects the generalisability of the results. Future investigations should attempt to include data collected from other locations with more widely-practiced bilingualism, such as Quebec or Catalonia. This may allow for an examination as to whether any particular language pairs exhibit especially strong effects, or whether language experience in general is the driving factor of the effects observed here. Additionally, there are a large number of general and language-based experiential factors which may affect both neuroimaging measures and cognitive performance^84^, which should be carefully considered and controlled for in future investigations into bilingual neuroadaptation which utilise heterogenous samples and continuous measures of experience. Despite these limitations, our novel task-driven resting-state approach provides a new tool through which to observe experience-based alterations to functional brain activity. The directional connectivity measure, non-linear statistical analysis, and measurement of bilingualism as a continuous experience used in the present study should also be considered by future studies to more clearly unravel the effects of bilingualism on the brain and cognition.

## Method

**Table 2:** Demographics of the sample of participants.

Variable	Mean	SD	Range
Age	33.11	9.57	18–57
Language count	3.18	0.94	2–5
L2 AoA	6.25	4.63	0–20
LSBQ composite	9.01	2.69	5.05–14.05

Variable	Levels	N	
Gender	Female	21	
	Male	7	
Education level	GCSE/O-level	1	
	A-level	4	
	Degree/Diploma	10	
	Postgraduate	13	
Ethnicity	Asian/Asian-British	6	
	Chilean	1	
	Hispanic	2	
	Latin American	1	
	Persian/Iranian	2	
	White	16	
Handedness	Right	26	
	Left	2	

**Table 3:** Self-reported languages, in order of fluency, spoken as L1, L2, L3, L4, and L5 by the sample of participants.

L1s	*N*	L2s	*N*	L3s	*N*	L4s	*N*	L5s	*N*
Bulgarian	1	Arabic	2	Albanian	1	Croatian	1	French	1
English	5	Badaga	1	Catalan	2	French	2	German	2
Finnish	1	English	22	English	1	Hindi	1	Malalayan	1
French	1	French	1	French	2	Italian	1		
German	2	Macedonian	1	Galician	1	Portuguese	1		
Greek	3	Spanish	1	Garhwali	1	Turkish	1		
Hindi	2			German	2				
Hungarian	1			Italian	4				
Italian	1			Portuguese	1				
Persian	3			Russian	1				
Polish	1			Serbian	2				
Spanish	7			Spanish	2				
				Tamil	1				
				Turkish	1				

### Participants

Recruitment targeted bilinguals who had been living in the UK for at least 3 years and spoke English as a non-native language, as previous research indicates that immersion in the L2 context drives neural adaptations^73^.

The following exclusion criteria were used during recruitment: must have been living in the UK for at least three years; no history of meningitis, encephalitis, head injury, or loss of consciousness; any vision impairment is corrected-to-normal. The total number of participants recruited was 32, with three participants being excluded due to a high number of artifacts in one or more EEG recordings, and an additional participant was excluded due to an outlying LSBQ composite score, making the final sample size 28 participants (Mean age = 33.11, SD = 9.57; female = 21). The language count of the sample ranged from 2 to 5 (Mean = 3.18, SD = 0.94), of which English was required to be one, and LSBQ composite scores, representing level of bilingualism, ranged from 5.05 to 14.05 (Mean = 9.01, SD = 2.69). Participant education level ranged from GCSE/O-level to Postgraduate (Master’s or PhD), with self-reported ethnicities including Asian/Asian-British, Chilean, Hispanic, Latin American, Persian/Iranian, and White. Demographics are reported fully in Table 2, and participants’ self-reported spoken languages in order of fluency in Table 3.

### Materials

#### Questionnaires

We collected demographic and language history data using the Language and Social Background Questionnaire (LSBQ)^72^, administered online using the Gorilla Experiment Builder^85^, and also contained the study’s information sheet and consent form.

#### Tasks

The data used in this study was collected as part of a larger project – we administered three tasks to each participant, with the presentation order counter-balanced. Each task was chosen from different domains which bilingualism is thought to affect. The procedure included a lexical retrieval task (language domain), a Flanker Inhibitory Control and Attention task (‘Flanker task’; attentional domain), and an AGL serial reaction-time task (hierarchical structure representation; domain-general). The details of and data produced from the language and attentional domain tasks are presented in Sheehan et al.^86^. Below is a description of the AGL serial reaction-time task used in this study.

*Artificial grammar-learning serial reaction-time task*^30,32^– *Domain-general*.

This task required participants to view serially presented stimuli on-screen representing the binary output of the Fib grammar. The 0s and 1s of the sequence are presented as red and blue circles respectively. The participant had to indicate which colour they just saw by using the keyboard – pressing ‘M’ for red (i.e., 0), and ‘Z’ for blue (i.e., 1). The first block consisted of a training sequence – the string used was not an output of the Fib grammar and contained multiple ungrammatical sub-sequences (e.g., 00 or 111). The order was pseudo-randomised and contained the same frequency of elements as the grammatical blocks and had a total length of 50 items. Experimental blocks followed, consisting of generation 11 of the Fibonacci grammar, which has a length of 144 symbols. Each experimental block contained a full generation of the grammar, with seven experimental blocks for a total of eight blocks. All stimuli were presented with an inter-stimulus interval of 500 ms. This task requires participants to implicitly process the transitional probabilities in the string and construct a hierarchical representation to predict which item would be presented next. Example stimuli from the task can be found in the Supplementary Materials.

#### Equipment

We used Brain Products equipment – a 32-electrode ActiCAP snap electrode set was connected to an electrode control box, and then to an amplifier, using a PowerPack to supply electricity^87^. The FCz electrode was used as the online reference, and data was recorded at a sampling rate of 500 Hz. We used E-Prime 3.0^88^ to present the tasks on a 24” monitor with a refresh rate of 60 Hz.

### Procedure

Prior to booking an appointment in the EEG lab, participants completed the LSBQ online in order to confirm their eligibility. As part of this questionnaire, informed consent was obtained from all participants after they had read and understood the study’s information sheet. Participants then attended the lab for EEG data collection. All recordings in this study were carried out in the same electromagnetically- and radio-frequency-shielded testing room in the Centre for Integrative Neuroscience and Neurodynamics at the University of Reading.

Before taking resting-state recordings, participants were asked to sit comfortably with their eyes closed for five minutes, to stay as still as possible, and to not fall asleep. The recordings were then started with BrainVision Recorder^89^. Following the first resting-state recording, the task-based stage of the experiment was verbally explained, and the task administration began. The task script was run with E-Prime^88^, and the participants were told that they could begin the task as soon as the testing room door was closed. Each task was designed to take roughly 20 min, after which a post-task five-minute resting-state recording was taken. This resulted in four resting-state recordings (1 x pre-task, 3 x post-task).

This procedure was carried out in accordance with all relevant guidelines and regulations, and received ethical approval and a favourable opinion for conduct from the University of Reading School of Psychology and Clinical Language Sciences Ethics Committee.

### Data processing & analysis

#### Pre-processing

Data was loaded into EEGLAB^90^ in order to re-reference offline using the Reference Electrode Standardisation Technique^91^. The re-referenced data was exported and loaded into BrainVision Analyzer^92^. In Analyzer, we applied high and low cut-off filters at 64 and 0.1 Hz and resampled the data to 128 Hz. We used a semi-automatic ocular correction ICA to check for and remove any eye-movement artifacts present. The recordings were then segmented into 10s segments with no overlap, and a semi-automatic artifact rejection procedure was applied. For this step, we used the following criteria for the automatic identification of artifacts within each segment: a gradient criterion which searches for a change of 10 µV per millisecond between two points, a min-max criterion which identifies a maximum difference in amplitude of 100 µV over 200 milliseconds, an amplitude criterion to find minimum amplitudes of -150 µV and maximums of 150 µV within each segment, and a low activity criterion to identify signals with activity lower than 0.5 µV over 100 milliseconds. Any segments containing artifacts not captured by the above criteria were manually selected and removed.

#### Functional connectivity analysis

**Fig. 5:**
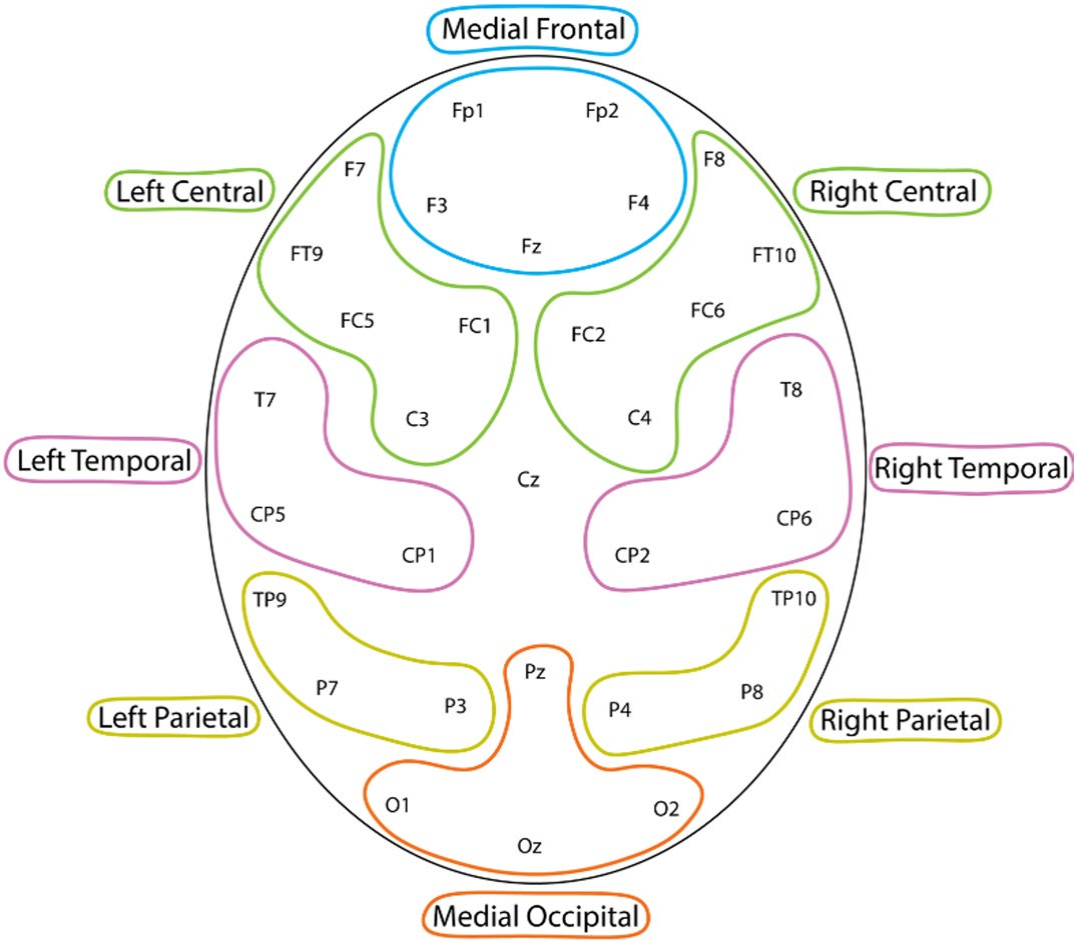
The electrode montage used for the functional connectivity analysis, forming our ROIs.

The pre-processed recordings were loaded into Brainstorm^93^, where, due to the low spatial resolution of EEG and to reduce computational demands, electrodes were grouped into a montage prior to Granger Causality estimation. The montage was designed to differentiate between activity in the frontal, central, temporal, and parietal regions, and can be seen on an electrode diagram in Fig. 5. In this step, the signals from each electrode grouped into a single region are averaged together, forming a normalised time series of the mean electrical signal received by the region.

The analysis was conducted in electrode space using the connectivity metric Granger Causality. This produced an 8 × 8 connectivity matrix for each participant in each condition (i.e., pre-task and post-task). The matrices were then exported as CSV files, converted to edge-lists, and transferred into a spreadsheet containing the corresponding demographic and language history data.

### Statistical analysis

The statistical programming language R^94^ and the RStudio IDE^95^ were used for our statistical analysis. Prior to importing the data into R, using the interquartile range (IQR) method, in which any data point which was below Q1–1.5 x IQR or above Q3 + 1.5 x IQR was considered an outlier, participant 30’s LSBQ composite score of 18.33 was identified as an outlier. This made the final sample size for statistical analysis 28 participants. Once imported, data from the participant with the outlying LSBQ composite score was removed, then ordinal and nominal variables were set to factors and contrast treatments applied. Generalised Additive Models using the ‘bam()’ function of the ‘mgcv’ package^74^ were chosen as the analysis method. Individual models were run for the pre- and post-task conditions. We modelled a smooth factor interaction term of LSBQ composite score with ROI-to-ROI connection, which fits a smooth for each of the 56 possible connections, against Granger Causality value. Both models contained the same covariates of age, L2 age of acquisition, participant education level (as a proxy for socioeconomic status^96^, gender, and a linear term for ROI-to-ROI connection (required for factor interactions), with random effects for participant number and order of task presentation included. Given that the sample of participants included only two left-handed individuals, we ran a nested version of the model described above, but additionally including participant handedness as a covariate. We then used a Chi Square test with the ‘anova.gam()’ function from the ‘mgcv’ package^74^ to conduct an approximate Likelihood Ratio Test, allowing us to determine whether the addition of handedness as a predictor significantly improves the fit of the model. For the pre-task model, the inclusion of handedness led to no significant improvement in model fit, and so the original model construction was chosen. For the post-task model, the Chi Square test indicated a trend towards significance which ultimately suggests that adding handedness did not improve the model fit. However, to be certain of this conclusion, we conducted another test using the Akaike Information Criterion (AIC)^97^, in which a lower score indicates a better model fit to the data. The AIC reflected the same conclusion; that the original model provided a better fit to the data, and so the original model was chosen. The code used for these models and checks can be found in the Supplementary Materials and the manuscript’s OSF repository, which is detailed in the Data Availability section.

Following the selection of the pre- and post-task models, multiple comparison corrections using the false discovery rate (FDR) method were applied to the results. Terms that remained significant for level of bilingualism following FDR correction were plotted using the ‘draw()’ function of the ‘gratia’ package^98^. To visualise the shared significant connection between conditions on a single plot, the ‘compare_smooths()’ function from the ‘gratia’ package was used with the ‘draw()’ function. The code used to run the models can be found in the Supplementary Materials, and the full R markdown can be found in an OSF repository which is provided in the Data Availability section.

### Assessment of model fit

The ‘gam.check()’ function of the ‘mgcv’ package^74^ was used in R to assess the fits of the significant models. Using the model structure described above necessitates each level of the interaction utilising the exact same number of basis functions (‘K-value’) to form each smooth, which can result in underfitting for some levels, and overfitting for others. To prevent this, we set the K-value of the interaction term in each model based on the degrees of freedom of the variable to allow a closer fit to the data. Due to the possibility of some connections exhibiting more non-linear relationships, and some with more linear relationships, we chose 14 basis functions (corresponding to a K-value of 15); half of the degrees of freedom of the main LSBQ composite score variable. To reduce the possibility of overfitting some levels of the interaction which may not require as many basis functions, a smoothing penalty was set at 0.02, restricting the possible ‘wiggliness’ of the smooth fitted to the data to avoid overfitting. Consequently, neither model exhibited any significant K-indexes.

## Supplementary Information

Below is the link to the electronic supplementary material.

Supplementary Material 1

## Data Availability

The data sheets, code, and task used for this manuscript are available in an Open Science Framework repository at https://osf.io/4qkfd/.
