# Supplementary material for: Bilingualism modulates functional connectivity induced by a domain-general artificial grammar learning task
Source: Sci Rep. 2026 Mar 9;16:12756. doi: 10.1038/s41598-026-42094-x (PMC13096162; doi:10.1038/s41598-026-42094-x)
Supplement: Supplementary file 1 — Supplementary Material 1 [file 41598_2026_42094_MOESM1_ESM.docx]

**Supplementary materials**

**1. Overview of L-grammar properties and how they differ from those used in typical AGL tasks**

Lindenmayer grammars significantly differ from the artificial grammars (AGs) typically used in AGL tasks in multiple ways; in terms of generative processes, structure, and complexity. Typical AGL task designs tend to rely on finite-state (generated linearly one symbol at a time; simple) or phrase-structure grammars (generated with rewrite rules which can include multiple items; more complex) to measure and assess statistical learning. However, L-grammars possess various properties which allow for more complex investigations into statistical learning involving hierarchical structure.

In terms of differences in generative properties (i.e. in the way each type of grammar is created, or generated), L-grammars and typical AGs each apply their rules and categorise their items differently. Firstly, in L-grammars, all rewrite rules are applied simultaneously, whereas in typical AGs, rules are applied sequentially and must follow a specific order – this is known as a ‘traffic convention’. L-grammars do not follow a traffic convention, and instead apply their rewrite rules to each item in an iteration at the same time in a top-down fashion. Secondly, typical AGs contain two categories of items: rewritable and non-rewritable (or non-terminal and terminal, respectively). A rewritable item is one which, when rewrite rules are applied, will generate an item or string according to the rule applied. A non-rewritable item is one which cannot be rewritten as another item or string – in conventional grammars, when a string consists of only non-rewritable items, the generation process ends. In contrast, L-grammars do not adopt these categories – each item in a string can be rewritten continuously, and so produces successive generations of strings for a theoretically infinite number of iterations.

These types of grammars also possess different structural qualities – that is, representational properties exhibited by the *output* of the grammars. L-grammars show unique structural properties which are not inherent to typical AGs. One of which is the property of self-similarity – a core characteristic of Lindenmayer grammars. This means that each generation of the grammar shows a degree of similarity with the previous, but without being identical. This is exemplified in the Fibonacci grammar (‘Fib’) by observing that each generation is the concatenation of the previous two generations. From generation 1 to 4, Fib generates as follows:

1

01

101

01101

This self-similarity creates a scale-free environment where the transitional probabilities between items in one generation are identical to the transitional probabilities between larger groups of items in the next generation. Linked to this property, L-grammars also exhibit hierarchy and recursive embedding. Each generation of an L-grammar can be thought of as a level of hierarchy, and so probabilistic (or ambiguous) transitions are recursively embedded into deterministic (or unambiguous/disambiguated) transitions at the next level. This particular property is what allows individuals to reconstruct non-adjacent hierarchical levels of the grammar by chunking the string into larger constituents, thus compressing the transitional probability information and bypassing the typical limits of human working memory. Finally, L-grammars also exhibit aperiodicity – their strings do not follow a periodic pattern, as typical AGs do. Whilst certain transitions in L-grammars are deterministic, a single linear function is unable to predict any specific point in the sequence. This means that L-grammars are more complex than those used in typical AGL tasks, and so are more closely aligned to the aperiodicity of natural language.

These properties allow for certain advantages in using L-grammars over typical AGs for cognitive tasks. Firstly, as L-grammars can be implemented in a serial reaction time design, they do not have to rely on grammaticality judgements to assess learning effects. Typical AGL designs first present the output of an AG to participants, and then probe them with a short sequence and ask whether it was generated by the same grammar as the longer sequence which they were just exposed to. This method relies on any implicit statistical learning which took place during exposure to the grammar becoming explicit (i.e. conscious) knowledge in order to respond to the grammaticality judgement. Consequently, this method is likely to lose sensitivity to the underlying implicit learning processes. Our implementation of L-grammars, however, does not require such explicit judgements, as implicit learning can be measured using changes in reaction times to the sequentially presented stimuli, remaining more sensitive to implicit learning without relying on the conversion of implicit knowledge to explicit knowledge. Secondly, whilst typical AGs can be predicted through the detection of recurring local patterns in the transitions between items, L-grammars cannot. Instead, for L-grammars, the hierarchical structure must be considered in order to resolve local ambiguous points and accurately predict subsequent items in the sequence. This allows researchers to separately probe sequential learning and hierarchical structure processing, and the interaction between the two. Finally, due to the nature of typical AG designs, which involve exposing a participant to the grammar’s output string, responses to grammaticality judgements depend on habituation to the target sequence in order to detect ungrammatical strings when presented. As L-grammars are aperiodic, habituation to the sequence would not provide any assistance in predicting subsequent items, as the pattern in the transitional probabilities shifts in different parts of the sequence. This prompts the brain to attempt a different approach based on hierarchical reconstruction instead of repetition.

In summary, L-grammars can be exploited as a method of measuring and assessing implicit statistical learning and hierarchical structure processing with high sensitivity in a way that typical AGL task designs cannot. Owing to their unique generative and representational properties detailed above, while L-grammars are not natural language grammars, L-grammar tasks are more closely linked to the complex processes involved in natural language processing. This means that they more accurately capture domain-general cognitive processes which may be affected by how much experience one has gained in applying these processes underpinning natural language use and bilingual experience.

**2. The full results of the behavioural performance analysis.**

Linear Mixed Models:

- Accuracy:

|  | Level of Ambiguity | | | | | | | | | | | |
| --- | --- | --- | --- | --- | --- | --- | --- | --- | --- | --- | --- | --- |
|  | 0 | | 1 | | 2 | | | 3 | | | 4 | |
| Accuracy: | *β* | *p* | *β* | *p* | | *β* | *p* | | *β* | *p* | *β* | *p* |
| Block | -0.001 |  | -0.017 | ****** | | -0.004 |  | | -0.038 | ****** | 0.001 |  |
| Ambiguity | 0.041 | ***** | 0.011 |  | | 0.007 |  | | -0.120 | ***** | -0.026 |  |
| LSBQ composite | -0.001 |  | -0.006 | ***** | | 0.001 |  | | -0.013 | ***** | -0.001 |  |
| Block * Ambiguity | 0.000 |  | 0.012 |  | | 0.005 |  | | 0.034 | ***** | -0.005 |  |
| Block * LSBQ composite | 0.000 |  | 0.002 | ****** | | 0.000 |  | | 0.003 | ***** | -0.001 |  |
| Ambiguity * LSBQ Composite | -0.001 |  | 0.004 |  | | -0.004 |  | | 0.010 |  | 0.001 |  |
| Block * Ambiguity * LSBQ Composite | 0.001 |  | -0.001 |  | | 0.000 |  | | -0.003 |  | 0.001 |  |

- Reaction time:

|  | Level of Ambiguity | | | | | | | | | |
| --- | --- | --- | --- | --- | --- | --- | --- | --- | --- | --- |
|  | 0 | | 1 | | 2 | | 3 | | 4 | |
| Reaction Time: | *β* | *p* | *β* | *p* | *β* | *p* | *β* | *p* | *β* | *p* |
| Block | -18.266 | ******* | -14.864 | ******* | -20.70 | ******* | -24.385 | ******* | -30.305 | ******* |
| Ambiguity | -1.92 |  | -37.254 | ***** | -37.603 | ***** | -37.533 |  | -73.195 | ****** |
| LSBQ composite | -11.861 | ****** | -9.277 |  | -14.616 | ******* | -11.336 | ***** | -17.791 | ******* |
| Block * Ambiguity | -22.669 | ******* | -12.213 | ****** | 4.180 |  | 14.978 | ***** | 14.866 |  |
| Block * LSBQ composite | 1.249 | ******* | 1.041 | ****** | 1.779 | ******* | 2.047 | ******* | 2.746 | ******* |
| Ambiguity * LSBQ Composite | -2.341 |  | 0.36 |  | 4.742 | ***** | 3.151 |  | 5.642 |  |
| Block * Ambiguity * LSBQ Composite | 1.271 | ******* | 0.253 |  | -0.875 |  | -1.569 | ***** | -1.53 |  |

Descriptives:

- By block:

|  | Accuracy | | Reaction Times (ms) | |
| --- | --- | --- | --- | --- |
| Block | Mean | SD | Mean | SD |
| 0 | 0.967 | 0.180 | 420.302 | 116.319 |
| 1 | 0.964 | 0.187 | 395.175 | 131.332 |
| 2 | 0.963 | 0.188 | 371.459 | 124.338 |
| 3 | 0.967 | 0.179 | 366.128 | 132.175 |
| 4 | 0.963 | 0.189 | 358.417 | 128.808 |
| 5 | 0.968 | 0.176 | 344.937 | 125.524 |
| 6 | 0.968 | 0.176 | 336.415 | 131.320 |
| Overall | 0.966 | 0.002 | 370.405 | 29.070 |
| Change | 0.001 | - | -83.888 | - |

- By level of ambiguity:

|  | Level of Ambiguity | | | | | | | | | |
| --- | --- | --- | --- | --- | --- | --- | --- | --- | --- | --- |
|  | 0 | | 1 | | 2 | | 3 | | 4 | |
| Accuracy: | Mean | SD | Mean | SD | Mean | SD | Mean | SD | Mean | SD |
| Overall | 0.969 | 0.173 | 0.960 | 0.195 | 0.935 | 0.246 | 0.926 | 0.262 | 0.941 | 0.236 |
| Ambiguous points | 0.935 | 0.246 | 0.926 | 0.262 | 0.946 | 0.225 | 0.924 | 0.265 | 0.953 | 0.211 |
| Disambiguated points | 0.990 | 0.100 | 0.982 | 0.135 | 0.928 | 0.258 | 0.927 | 0.260 | 0.936 | 0.245 |
| Overall change | -0.007 | 0.059 | 0.005 | 0.062 | -0.035 | 0.095 | -0.009 | 0.109 | -0.037 | 0.138 |
| Ambiguous points change | -0.033 | 0.069 | 0.000 | 0.081 | -0.046 | 0.109 | -0.045 | 0.119 | -0.024 | 0.167 |
| Disambiguated points change | 0.018 | 0.032 | 0.009 | 0.034 | -0.025 | 0.079 | 0.028 | 0.086 | -0.049 | 0.104 |
|  | 0 | | 1 | | 2 | | 3 | | 4 | |
| Reaction Time (ms): | Mean | SD | Mean | SD | Mean | SD | Mean | SD | Mean | SD |
| Overall | 359.004 | 127.093 | 388.973 | 132.601 | 395.093 | 114.265 | 429.906 | 127.496 | 399.707 | 113.186 |
| Ambiguous points | 395.093 | 114.265 | 429.906 | 127.496 | 398.687 | 114.222 | 434.778 | 123.774 | 413.827 | 126.876 |
| Disambiguated points | 337.923 | 129.450 | 365.121 | 129.670 | 392.826 | 114.251 | 426.917 | 129.664 | 394.176 | 106.873 |
| Overall change | -78.560 | 68.229 | -65.140 | 64.952 | -36.781 | 55.010 | -31.627 | 68.687 | -28.454 | 63.674 |
| Ambiguous points change | -40.232 | 44.696 | -33.380 | 59.973 | -25.242 | 55.245 | -27.040 | 60.960 | -34.264 | 68.743 |
| Disambiguated points change | -116.887 | 66.680 | -96.900 | 53.991 | -48.319 | 53.375 | -36.215 | 76.632 | -22.644 | 59.006 |

**3. Plots visualising significant terms from the behavioural performance analysis:**

Reaction times:

- Ambiguity Level 0:


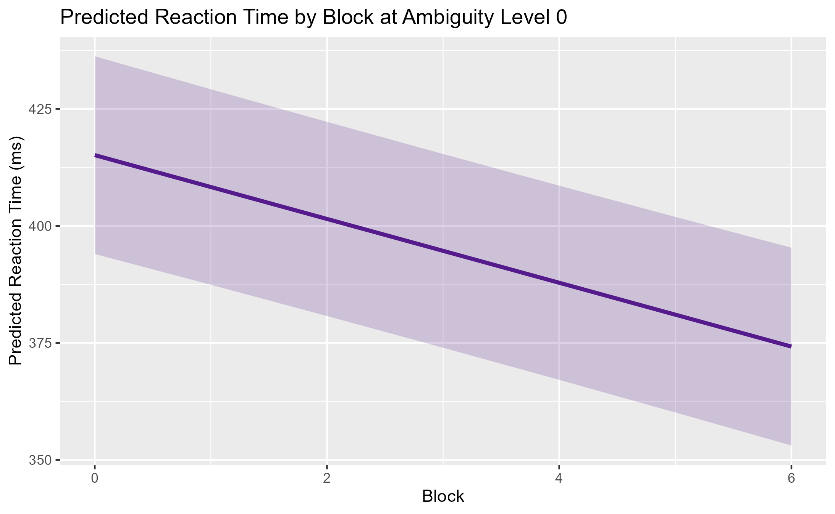


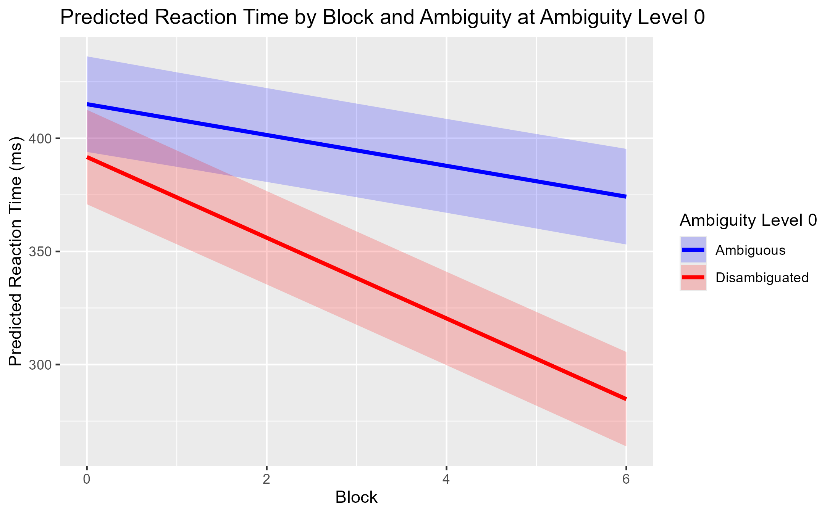


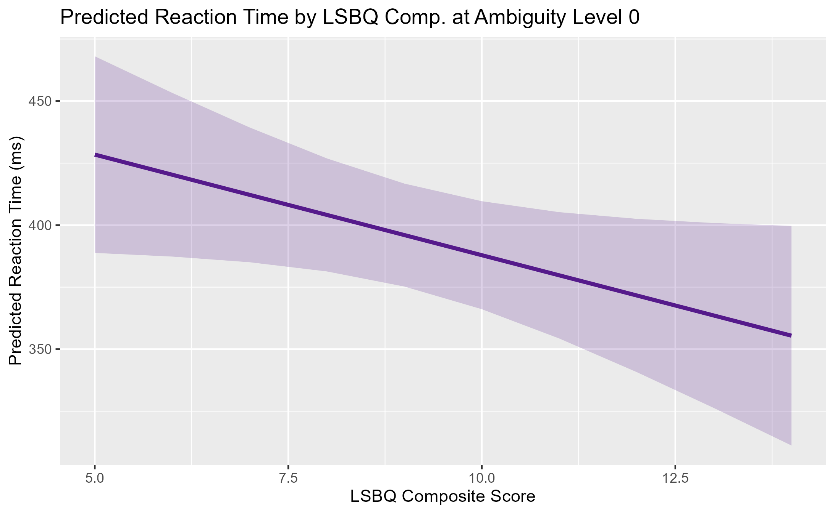


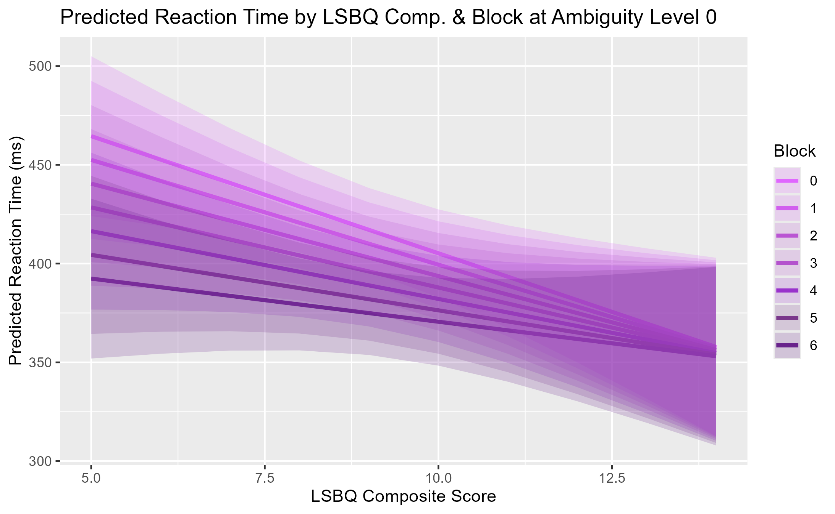


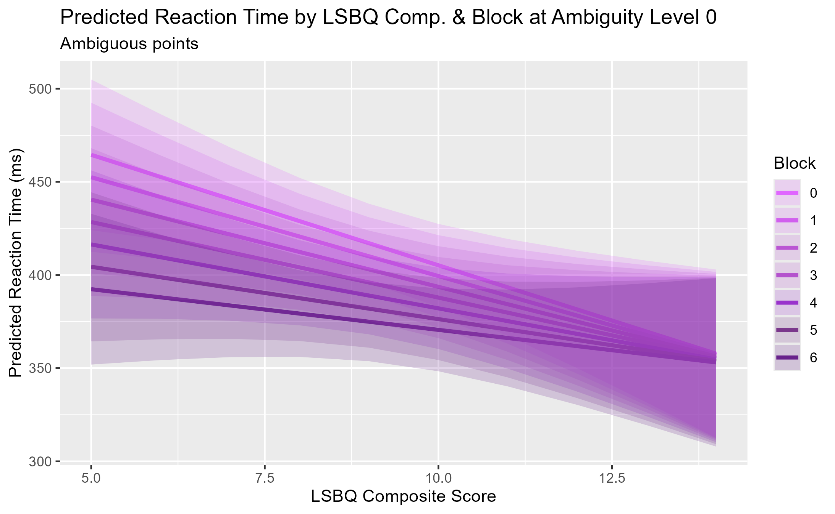


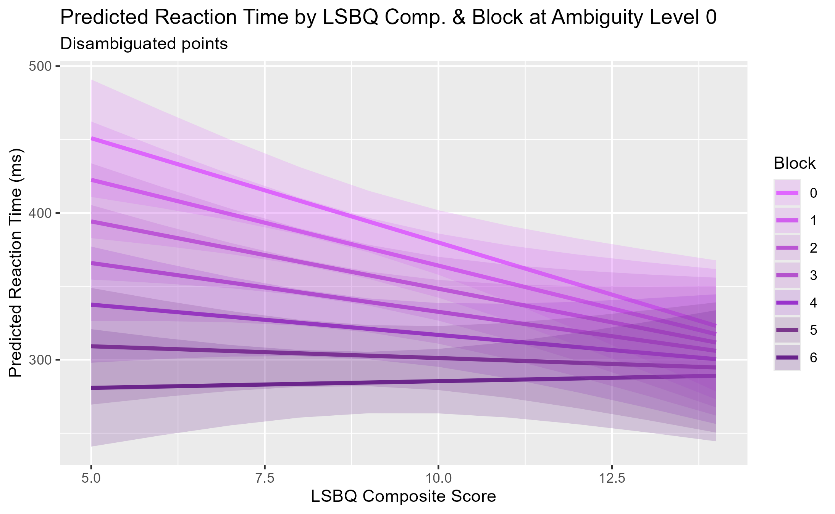


- Ambiguity Level 1:


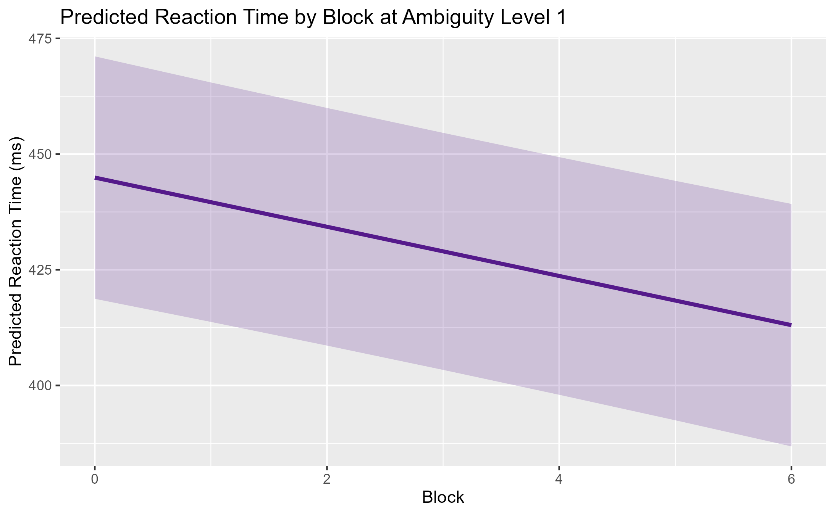


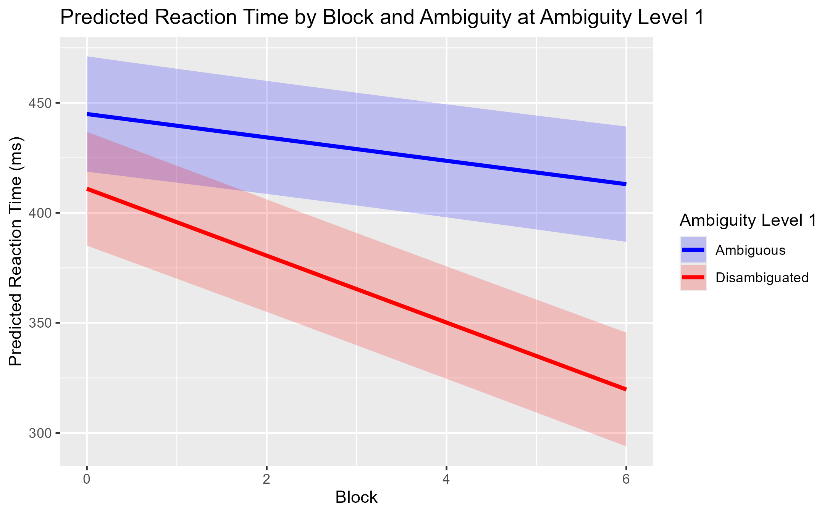


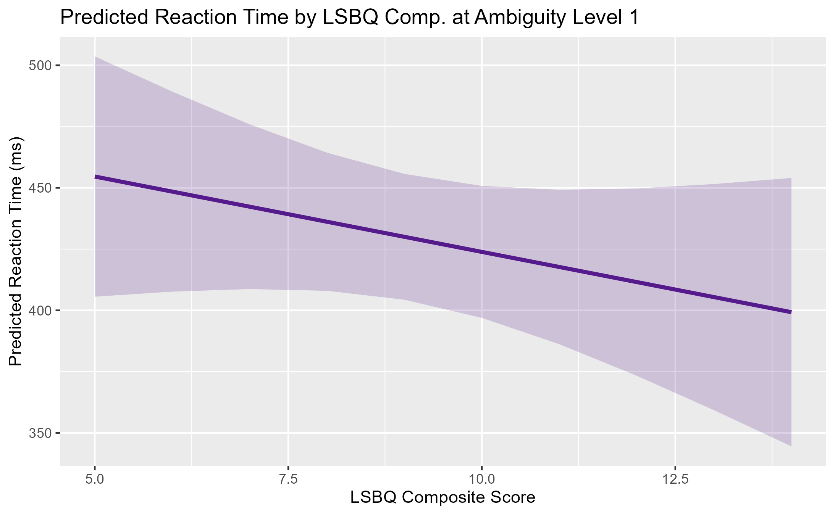


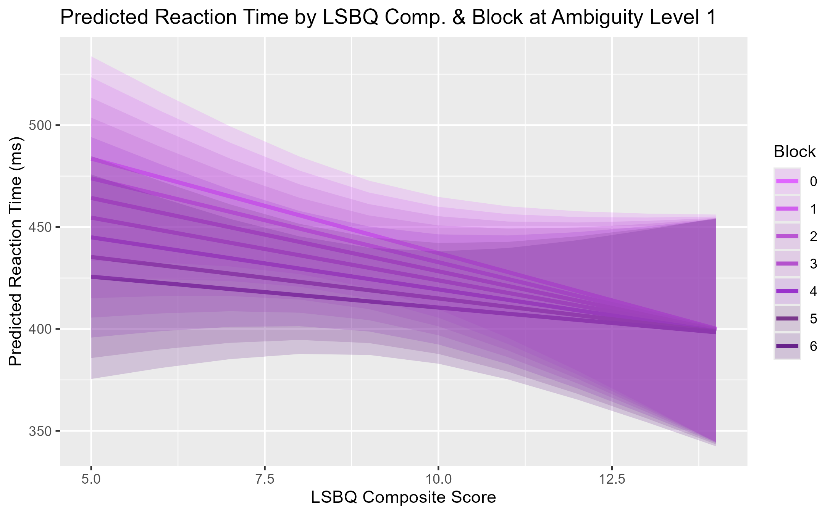


- Ambiguity Level 2:


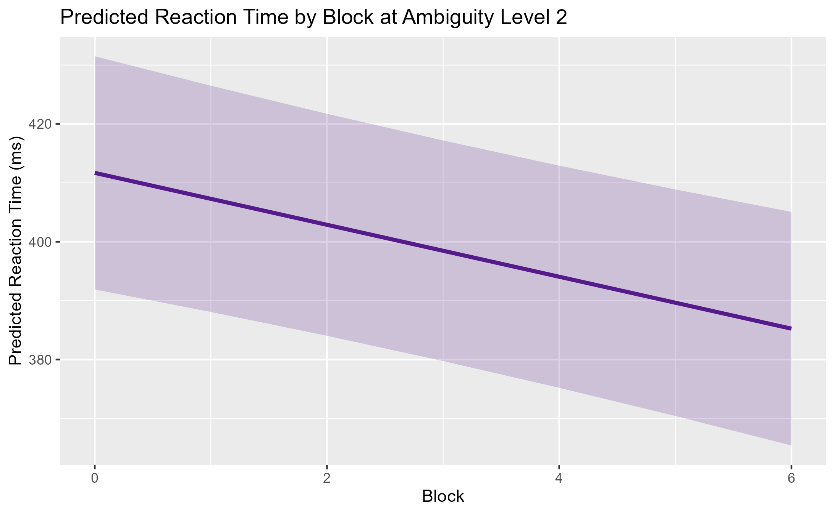


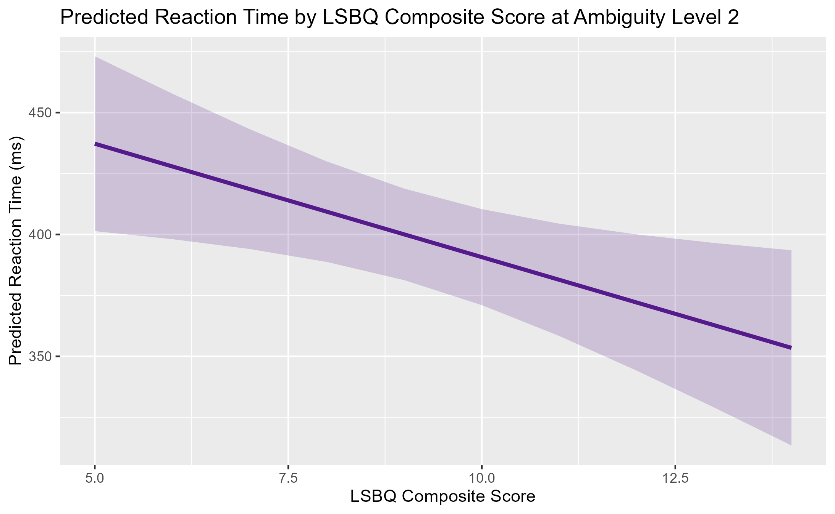


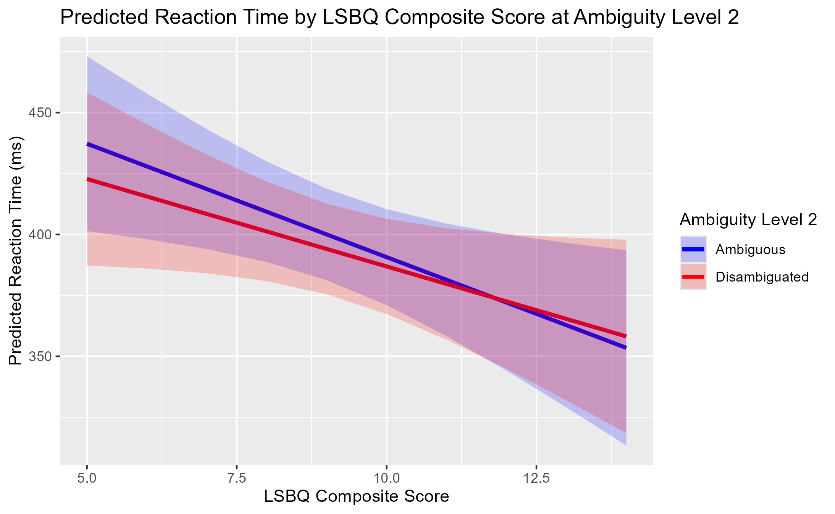


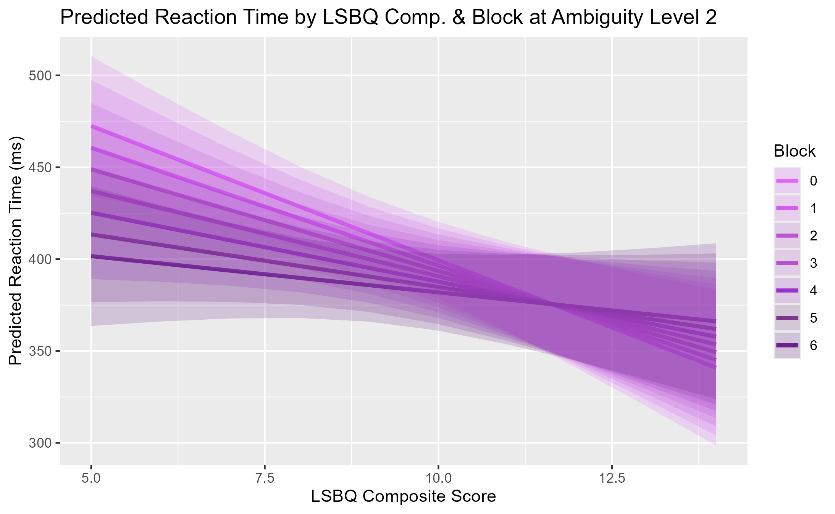


- Ambiguity Level 3:


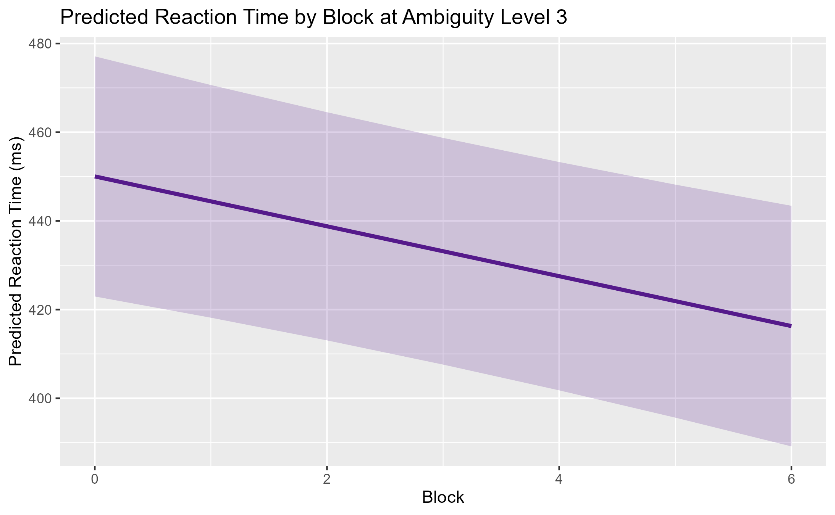


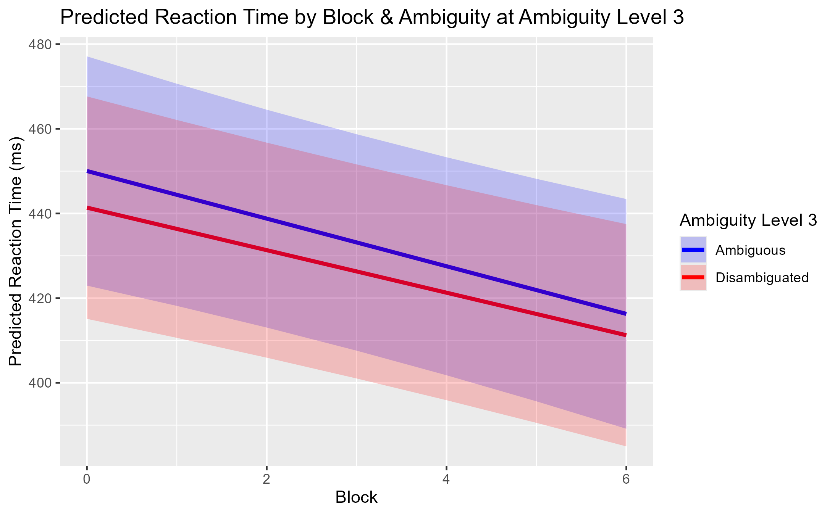


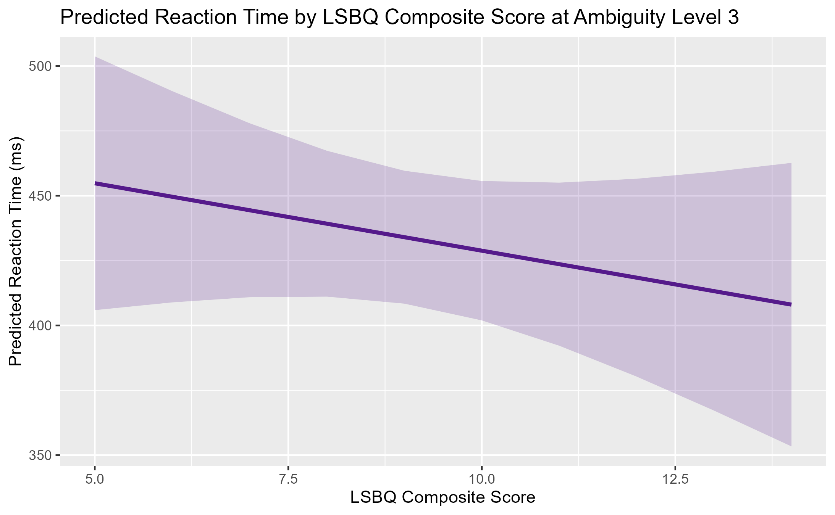


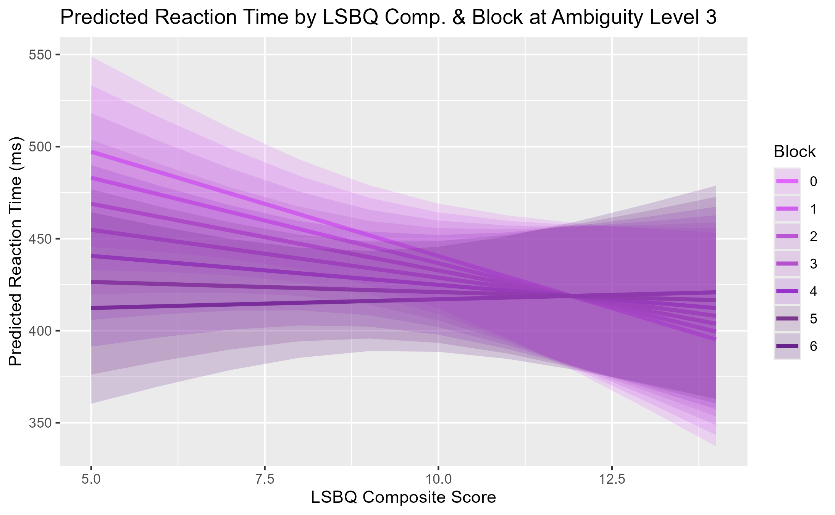


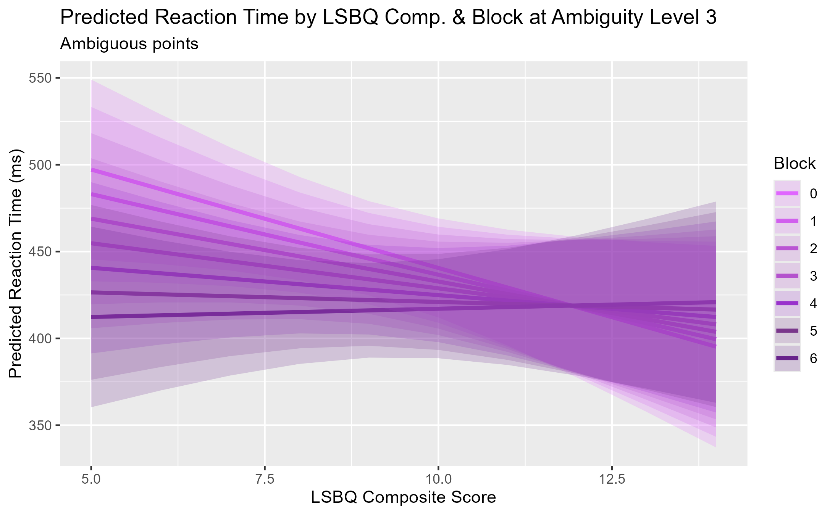


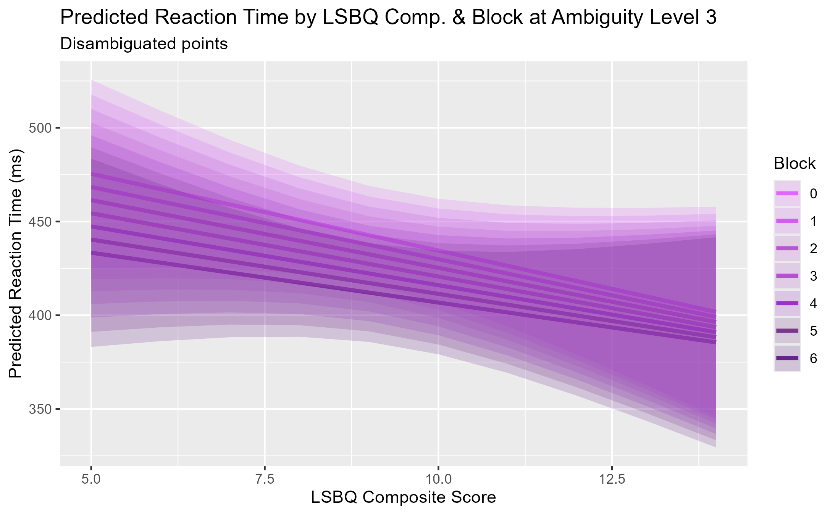


- Ambiguity Level 4:


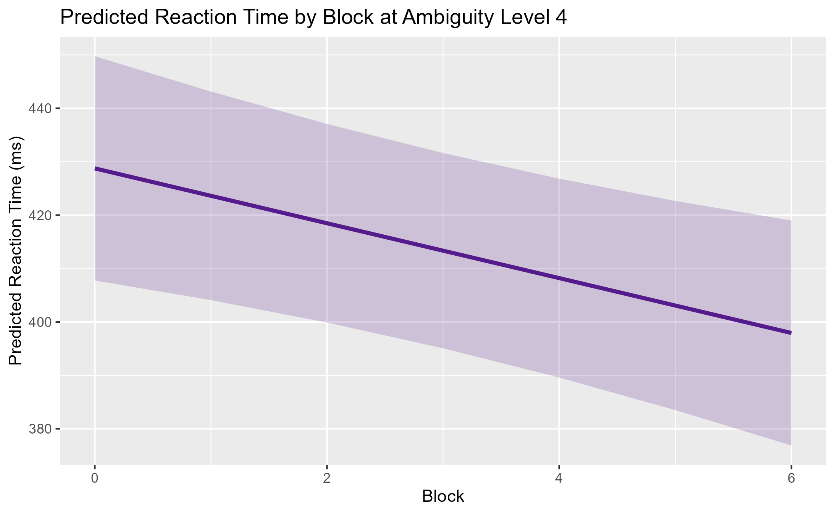


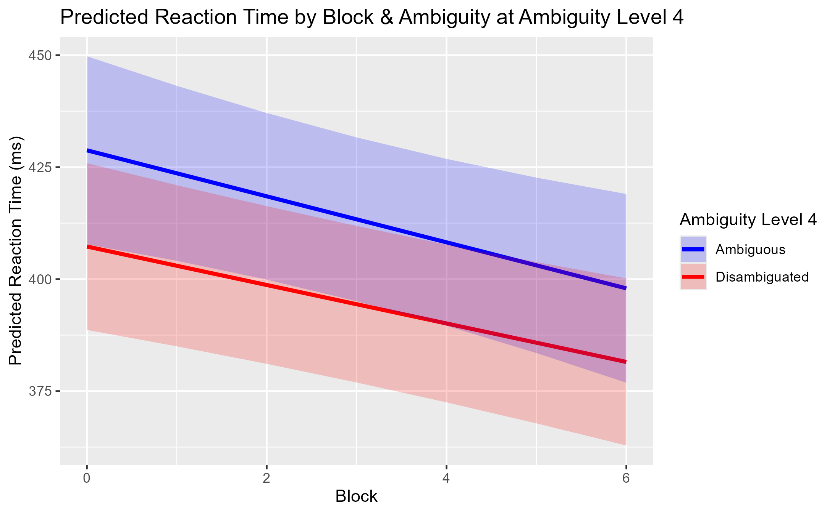


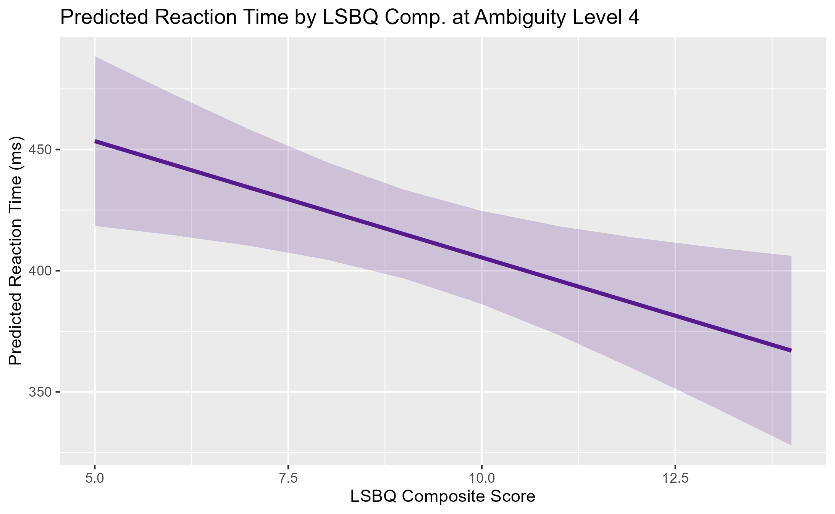


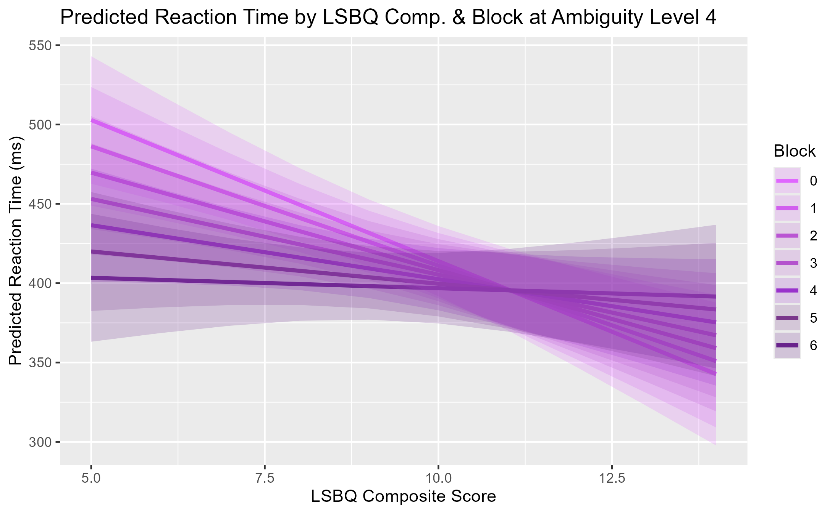


Accuracy:

- Ambiguity Level 1:


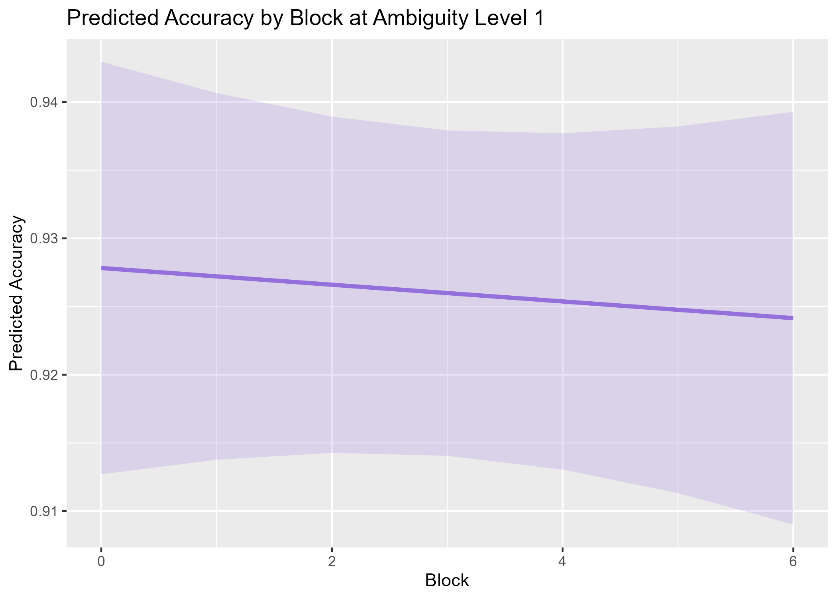


**
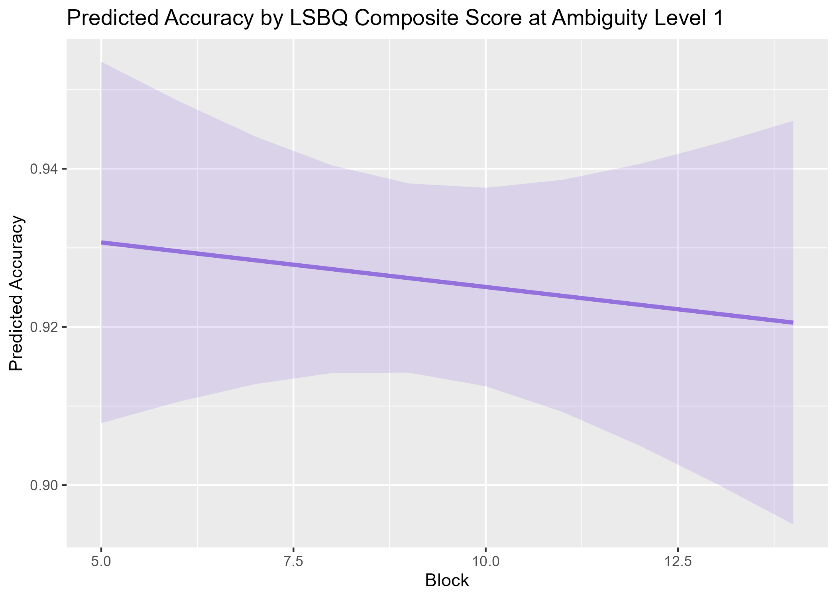
**

**
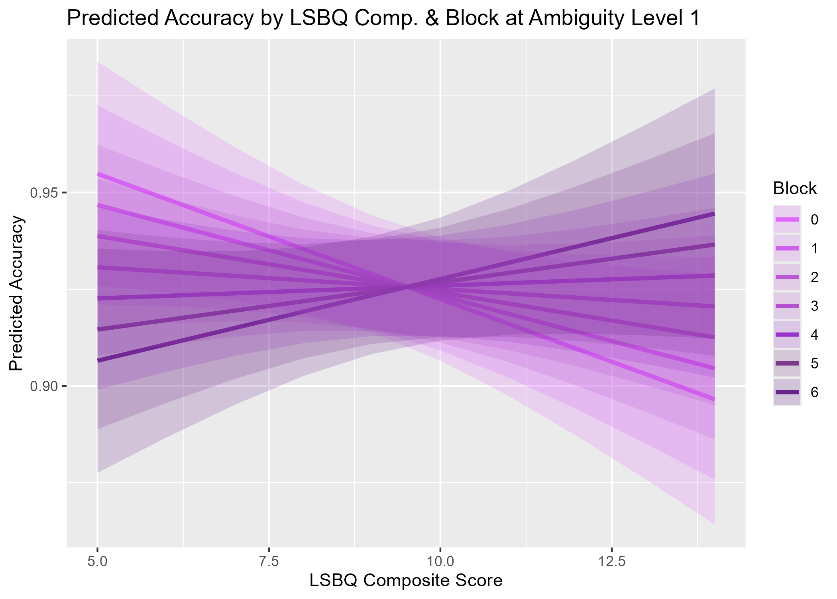
**

- Ambiguity Level 3:


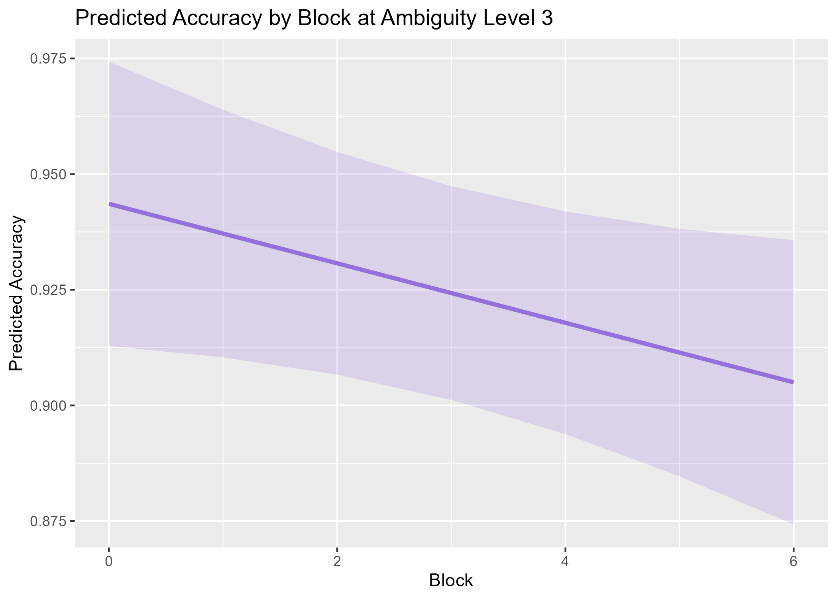


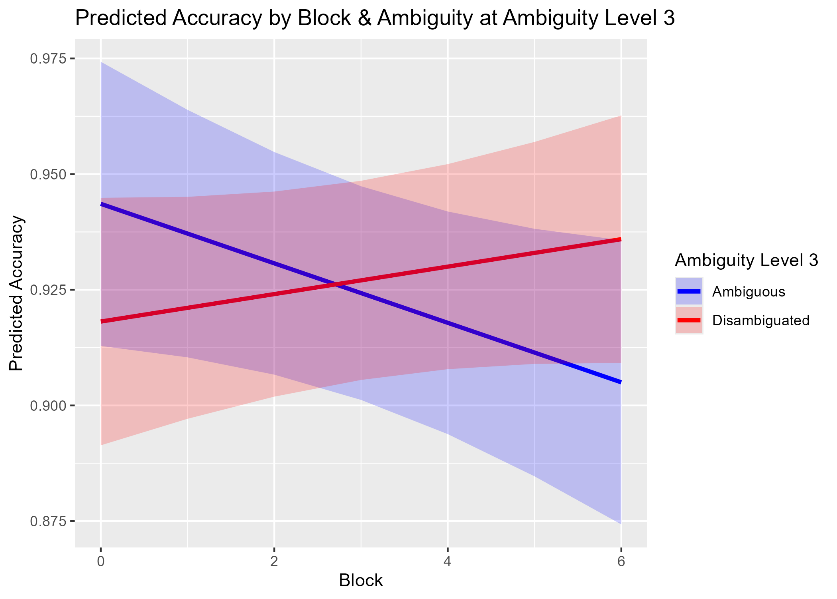


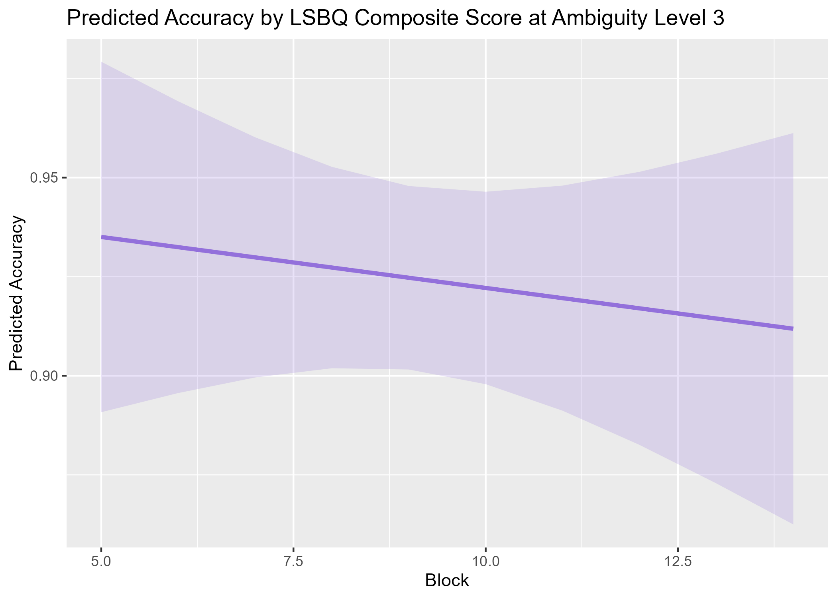


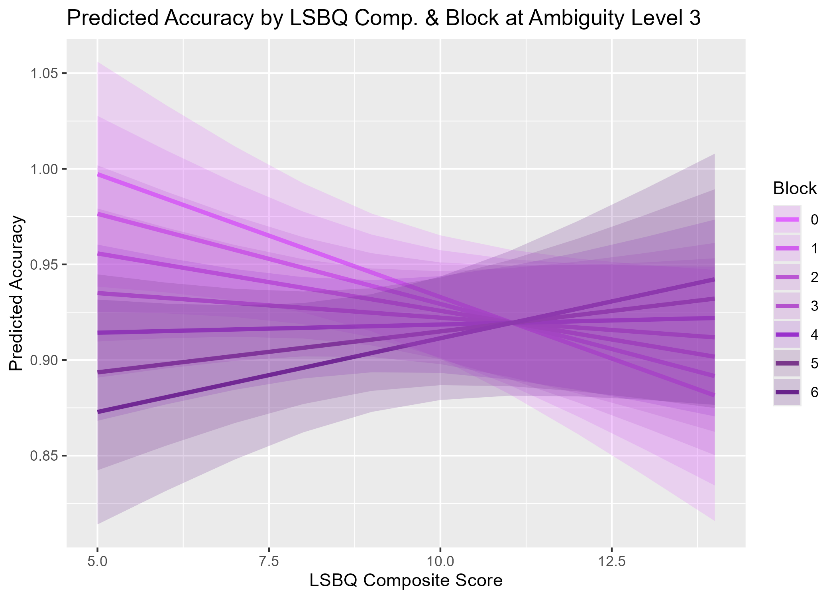


**4. Example stimuli from the Fibonacci grammar serial reaction time task**

Showing the corresponding visual items for the 0s (red) and 1s (blue) of the output string.


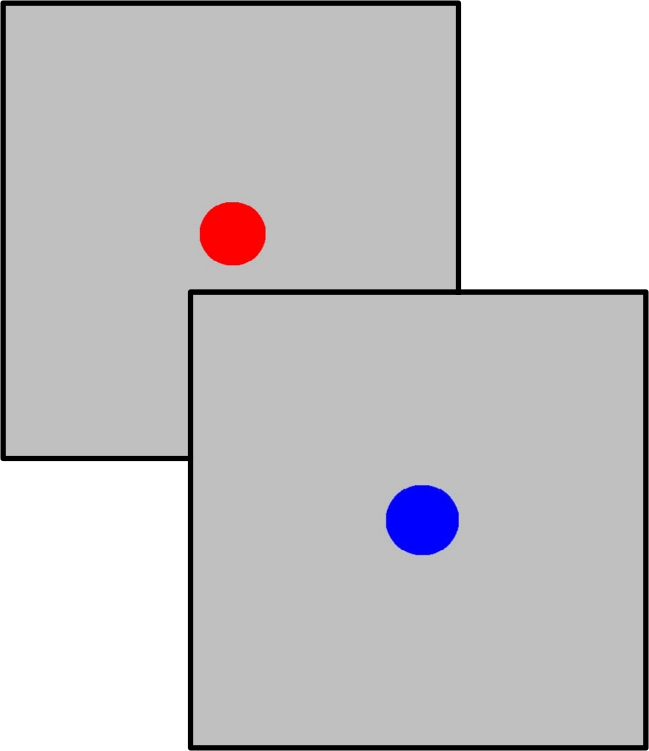


**5. Code used to run the models in R.**

*Pre-task:*

lsbq_pretask <- bam(connectivity ~

s(lsbq_comp, by = region, k = 15, sp = 0.02) +

region +

s(taskorder, bs = "re") +

s(subjid, bs = "re") +

age + lang2_aoa + education_subj_quant + gender_quant,

data = projectdata_long %>%

filter(condition == "pretask"),

method = "fREML", discrete = TRUE, nthreads = 21)

*Post-task:*

lsbq_posttask <- bam(connectivity ~

s(lsbq_comp, by = region, k = 15, sp = 0.02) +

region +

s(taskorder, bs = "re") +

s(subjid, bs = "re") +

age + lang2_aoa + education_subj_quant + gender_quant,

data = projectdata_long %>%

filter(condition == "postfib"),

method = "fREML", discrete = TRUE, nthreads = 21)

**6. Code used to run the supplementary models & comparisons in R.**

*Pre-task:*

# Fit model with handedness added

lsbq_pretask_h <- bam(connectivity ~

s(lsbq_comp, by = region, k = 15, sp = 0.02) +

region +

s(taskorder, bs = "re") +

s(subjid, bs = "re") +

age + lang2_aoa + education_subj_quant + gender_quant + handedness_quant,

data = projectdata_long %>%

filter(condition == "pretask"),

method = "fREML", discrete = TRUE, nthreads = 21)

# Compare to original model

anova.gam(lsbq_pretask, lsbq_pretask_h, test = "Chisq")

*Post-task:*

# Fit model with handedness added

lsbq_posttask_h <- bam(connectivity ~

s(lsbq_comp, by = region, k = 15, sp = 0.02) +

region +

s(taskorder, bs = "re") +

s(subjid, bs = "re") +

age + lang2_aoa + education_subj_quant + gender_quant + handedness_quant,

data = projectdata_long %>%

filter(condition == "postfib"),

method = "fREML", discrete = TRUE, nthreads = 21)

# Compare to original model

anova.gam(lsbq_posttask, lsbq_posttask_h, test = "Chisq")

# Further check

AIC(lsbq_posttask, lsbq_posttask_h)

# Fit model with task order as fixed effect instead of random effect

lsbq_posttask_t <- bam(connectivity ~

s(lsbq_comp, by = region, k = 15, sp = 0.02) +

region +

s(subjid, bs = "re") +

age + lang2_aoa + education_subj_quant + gender_quant + taskorder,

data = projectdata_long %>%

filter(condition == "postfib"),

method = "fREML", discrete = TRUE, nthreads = 21)

# Check significance of ‘taskorder’ levels

summary(lsbq_posttask_t)

# Compare to original model

AIC(lsbq_posttask, lsbq_posttask_t)
